# Demystifying the impact of prenatal tobacco exposure on the placental immune microenvironment: Avoiding the tragedy of mending the fold after death

**DOI:** 10.1111/jcmm.17846

**Published:** 2023-09-12

**Authors:** Xiaoxuan Zhao, Yuepeng Jiang, Xiao Ma, Qujia Yang, Xinyi Ding, Hanzhi Wang, Xintong Yao, Linxi Jin, Qin Zhang

**Affiliations:** ^1^ Department of Traditional Chinese Medicine (TCM) Gynecology Hangzhou TCM Hospital Affiliated to Zhejiang Chinese Medical University Hangzhou China; ^2^ Research Institute of Women's Reproductive Health Zhejiang Chinese Medical University Hangzhou China; ^3^ Zhejiang Chinese Medical University Hangzhou China

**Keywords:** bioinformatics analysis, immune landscape, machine learning, placenta, prenatal tobacco exposure, treatment

## Abstract

Prenatal tobacco exposure (PTE) correlates significantly with a surge in adverse pregnancy outcomes, yet its pathological mechanisms remain partially unexplored. This study aims to meticulously examine the repercussions of PTE on placental immune landscapes, employing a coordinated research methodology encompassing bioinformatics, machine learning and animal studies. Concurrently, it aims to screen biomarkers and potential compounds that could sensitively indicate and mitigate placental immune disorders. In the course of this research, two gene expression omnibus (GEO) microarrays, namely GSE27272 and GSE7434, were included. Gene set enrichment analysis (GSEA) and immune enrichment investigations on differentially expressed genes (DEGs) indicated that PTE might perturb numerous innate or adaptive immune‐related biological processes. A cohort of 52 immune‐associated DEGs was acquired by cross‐referencing the DEGs with gene sets derived from the ImmPort database. A protein–protein interaction (PPI) network was subsequently established, from which 10 hub genes were extracted using the maximal clique centrality (MCC) algorithm (*JUN, NPY, SST, FLT4, FGF13, HBEGF, NR0B2, AREG, NR1I2, SEMA5B*). Moreover, we substantiated the elevated affinity of tobacco reproductive toxicants, specifically nicotine and nitrosamine, with hub genes through molecular docking (*JUN, FGF13* and *NR1I2*). This suggested that these genes could potentially serve as crucial loci for tobacco's influence on the placental immune microenvironment. To further elucidate the immune microenvironment landscape, consistent clustering analysis was conducted, yielding three subtypes, where the abundance of follicular helper T cells (*p* < 0.05) in subtype A, M2 macrophages (*p* < 0.01), neutrophils (*p* < 0.05) in subtype B and CD8+ T cells (*p* < 0.05), resting NK cells (*p* < 0.05), M2 macrophages (*p* < 0.05) in subtype C were significantly different from the control group. Additionally, three pivotal modules, designated as red, blue and green, were identified, each bearing a close association with differentially infiltrated immunocytes, as discerned by the weighted gene co‐expression network analysis (WGCNA). Functional enrichment analysis was subsequently conducted on these modules. To further probe into the mechanisms by which immune‐associated DEGs are implicated in intercellular communication, 20 genes serving as ligands or receptors and connected to differentially infiltrating immunocytes were isolated. Employing a variety of machine learning techniques, including one‐way logistic regression, LASSO regression, random forest and artificial neural networks, we screened 11 signature genes from the intersection of immune‐associated DEGs and secretory protein‐encoding genes derived from the Human Protein Atlas. Notably, CCL18 and IFNA4 emerged as prospective peripheral blood markers capable of identifying PTE‐induced immune disorders. These markers demonstrated impressive predictive power, as indicated by the area under the curve (AUC) of 0.713 (0.548–0.857) and 0.780 (0.618–0.914), respectively. Furthermore, we predicted 34 potential compounds, including cyclosporine, oestrogen and so on, which may engage with hub genes and attenuate immune disorders instigated by PTE. The diagnostic performance of these biomarkers, alongside the interventional effect of cyclosporine, was further corroborated in animal studies via ELISA, Western blot and immunofluorescence assays. In summary, this study identifies a disturbance in the placental immune landscape, a secondary effect of PTE, which may underlie multiple pregnancy complications. Importantly, our research contributes to the noninvasive and timely detection of PTE‐induced placental immune disorders, while also offering innovative therapeutic strategies for their treatment.

## 1 INTRODUCTION

There are more than 7000 chemical compounds in cigarettes, including numerous recognized reproductive toxins, which consistently inflict detrimental impacts on pregnancy outcomes,^1^ such as miscarriage, preterm birth, stillbirth and preeclampsia.^2^, ^3^, ^4^ Despite the wealth of evidence highlighting these risks, the prevalence of PTE among women remains concerning. Statistically, PTE is particularly widespread among women in socioeconomically disadvantaged countries. According to a representative national survey conducted in France, a mere 46% of women who smoked prior to pregnancy managed to abstain during gestation, leaving a significant 54% persisting with their harmful smoking habits.^5^ Furthermore, in South Africa, an alarming 56.3% of women have been reported to smoke during pregnancy.^6^ Consequently, the prevalence of PTE remains a significant public health concern and requires close attention.

The placenta, a temporary mammalian organ that bridges the maternal and foetal circulatory systems, is a primary target of tobacco exposure. Besides fulfilling several roles such as serving as a barrier, facilitating substance exchange and secreting hormones, the placenta acts as a critical immune regulator.^7^ More importantly, the placenta is also an influential immune regulator, it establishes a delicate balance between tolerating the allogeneic embryo and defending against external stimuli, thereby cultivating an immune microenvironment conducive to foetal growth and development.^8^ Additionally, reprogramming of gene expression and intermolecular interactions in the placenta as a result of exposure to risk factors may adversely affect immune modulatory function, thereby leading to multiple pregnancy complications. There is evidence that tobacco can adversely affect both intrinsic and adaptive immunity.^9^, ^10^, ^11^ Nevertheless, our understanding of the precise molecular mechanisms through which PTE detrimentally affects the placental immune microenvironment remains incomplete, owing to the intricate makeup of tobacco, the plethora of potential targets and the constraints inherent in traditional experimental approaches. Additionally, the dependency on retrospective studies analysing postnatal samples for identifying PTE‐induced pathological damage drastically hampers the prompt deployment of effective strategies. Therefore, the development and implementation of innovative research methodologies are of utmost urgency to unravel the distinct mechanisms leading to placental immunity disturbances due to PTE, enabling early identification of disorders and the timely initiation of preventive measures.

Recent advances in microarray technology and bioinformatics have substantially enriched our understanding of pathological mechanisms and facilitated the precise identification of candidate targets for drug design.^12^ Machine learning, with its robust classification capabilities, is increasingly utilized to learn high‐dimensional gene expression data. The synergistic integration of bioinformatics analysis and machine learning offers a novel and vital approach for identity biomarkers and deciphering pathological mechanisms at the molecular level, resonating with the latest research trends. Nevertheless, the application of this method remains scarce in studies of placental disorders resulting from PTE, suggesting a wealth of untapped potential information awaiting exploration.

**FIGURE 1:**
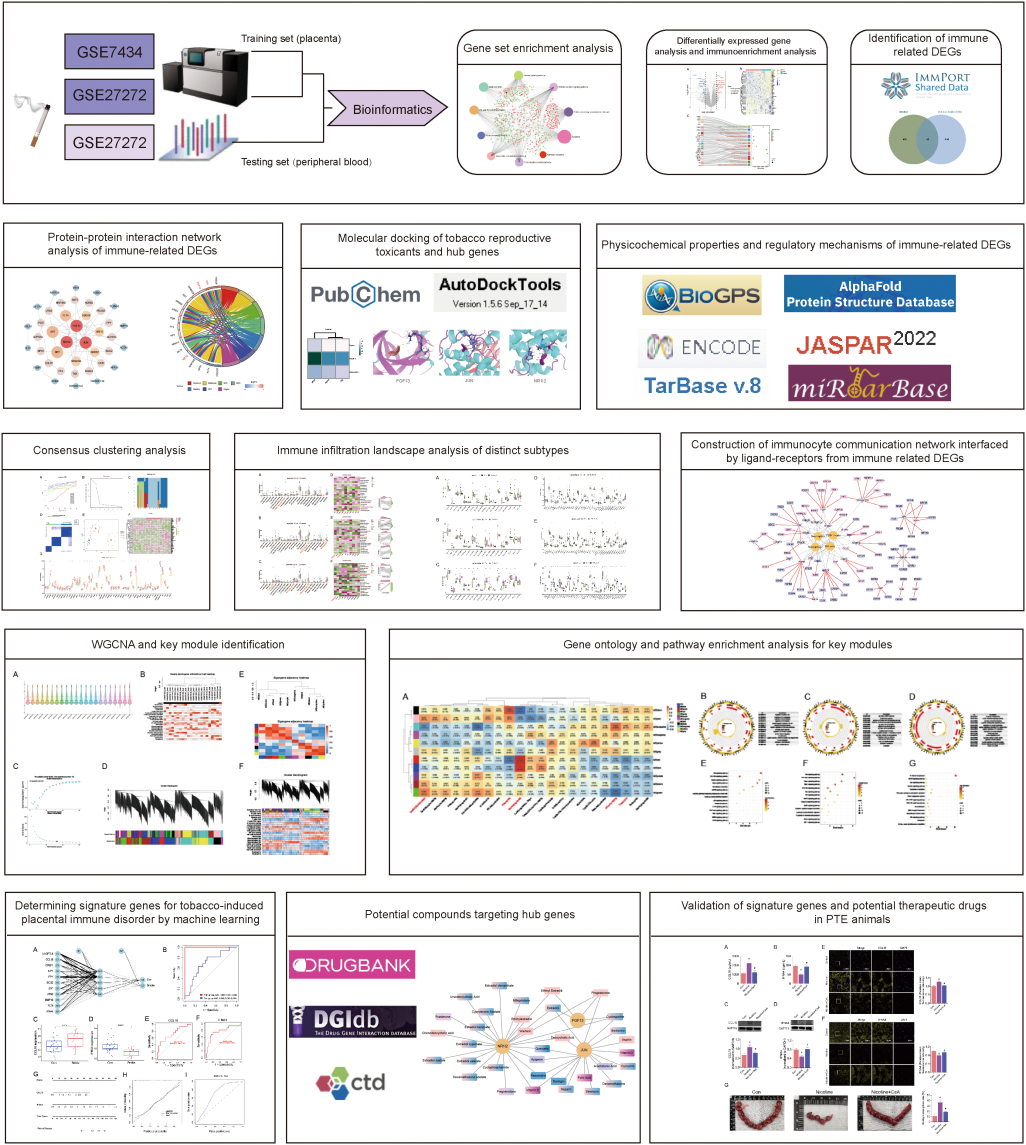
The flow diagram of the study.

In this study, microarrays concerning PTE from placenta and peripheral blood were sourced from the GEO database. This allowed us to examine the impact of PTE on the placental immune microenvironment, identify sensitive biomarkers within peripheral blood that could noninvasively indicate placental immune damage, and pinpoint potential therapeutic compounds. Initially, a GSEA and immune enrichment scrutiny were conducted on DEGs to elucidate the intimate link between PTE and altered immunological attributes in the placenta. Subsequently, the focus shifted to immune‐related DEGs, from which a PPI network was constructed, and hub genes were identified. This led to the exploration of tissue distribution, physicochemical properties and the expression regulation network of the aforementioned hub genes. Additionally, molecular docking was undertaken to assess the affinity between hub genes and representative reproductive toxicants found in tobacco. Moreover, a consensus clustering analysis was performed predicated on hub genes to ascertain the immune infiltration attributes of distinct subtypes. Following this, a WGCNA was employed to identify modules intimately associated with differentially infiltrated immunocytes, with functional enrichment analysis carried out on genes within key modules. Furthermore, visualization of the intercellular communication network among differentially infiltrating immunocytes was accomplished. Regarding the screening of signature biomarkers, machine learning was deployed to identify secretory sensitive markers from immune‐related genes. Notably, these signature genes were validated in gene sets containing peripheral blood samples from PTE or non‐PTE women. Also, we uncovered 34 active compounds either backed by empirical evidence or approved by the Food and Drug Administration (FDA) that could interact with representative hub genes, potentially beneficial during pregnancy. Lastly, animal experiments were conducted to corroborate changes in signature genes attributed to PTE and to determine the intervention effect of potential compounds on these genes. The comprehensive workflow diagram is depicted in Figure 1.

## 2 MATERIALS AND METHODS

### 2.1 Microarray datasets acquisition

Microarray data were acquired from the National Center for Biotechnology Information (NCBI) GEO database (https://www.ncbi.nlm.nih.gov/geo/),^13^ with the searching word of ‘smoking’, ‘prenatal’, ‘tobacco’ and ‘pregnancy’. Gene sets that met the following conditions could be included: (1) The research type was array‐based expression; (2) the species was set to homo sapiens; (3) the samples were from women with or without PTE; and (4) each group contained a minimum of five samples. According to the above criteria, two microarray datasets were included: GSE27272 based on GPL6883 (Illumina HumanRef‐8 v3.0 expression beadchip) platform and GSE7434 based on GPL570 (Affymetrix Human Genome U133 Plus 2.0 Array) platform. GSE7434 contained 10 placental samples, of which five were PTE and five were tobacco‐free, and GSE27272 contained placental tissue from 17 PTE women and 37 tobacco‐free women, as well as peripheral blood samples from 16 PTE and 35 tobacco‐free women (Table 1). Then, the relevant matrix files were downloaded for further analysis. Since sample data for this study were freely available from public sources, no patient consent or ethics committee approval was required.

**TABLE 1:** The basic information of included dataset.

GSE no.	No. of samples	Platform	Description	Samples	Country	References	Type
GSE7434	5 vs. 5	GPL570 Affymetrix Human Genome U133 Plus 2.0 Array	Gene expression in placenta from five smoking and five non‐smoking mothers analysed by Affymetrix Human Genome U133 Plus 2.0 Array	Placenta	Finland	PMID: 17928820	Training set
GSE27272	37 vs. 17	GPL6883 Illumina HumanRef‐8 v3.0 expression beadchip	Placental tissue from 17 prenatal tobacco‐exposure and 37 tobacco‐free women	Placenta	Czech Republic	PMID: 21803418	Training set
GSE27272	35 vs. 16	–	Peripheral blood samples from 16 prenatal tobacco‐exposed and 35 tobacco‐free women	Maternal peripheral blood	–	–	Testing set

### 2.2 Data preprocessing and normalization

The raw data were preprocessed and normalized by R statistical software (version 4.1.2, https://www.r‐project.org/) and Bioconductor analysis tools (http://www.bioconductor.org/). The ‘affy’ R language package was applied to conduct robust multi‐array averaging (RMA) background correction, complete log_2_ transformation.^14^ When multiple probes corresponded to one specific gene, their average expression level was considered.^15^ The probes were annotated with annotation files, and probes without corresponding gene symbols were removed. Moreover, we used the surrogate variable analysis in Bioconductor to reduce batch effects and other variables.^16^ Finally, the principal component analysis (PCA) before and after batch correction was shown in Figure S1.

### 2.3 GSEA associated with PTE

GSEA is a statistical method for analysing genome‐wide expression profile microarray data that allows the ranking of predefined genes according to the degree of differential expression in two types of samples to determine whether there are statistically significant differences in the expression of predefined gene sets.^17^, ^18^ In this study, to ascertain the status of immune‐related pathways on account of PTE, GSEA was performed based on KEGG signalling pathway gene sets assessed from the MSigDB (http://www.gsea‐msigdb.org/gsea/msigdb/index.jsp). Permutations were set to 10,000 to obtain normalized enrichment score (NES) in GSEA. Gene sets with *p* < 0.05 were considered significantly enriched.

### 2.4 Differentially expressed gene analysis and immunoenrichment analysis

After that, DEGs were screened out by using the ‘limma’ package, with the filtering condition of *p* value <0.05 and |log_2_FC| > 0.1. Besides, the volcano map and heat map of DEGs were, respectively, drawn by the ‘ggplot2’ package and the ‘pheatmap’ package. Then, the ClueGO plug‐in in Cytoscape (version 3.7.1, http://www.cytoscape.org/)^19^ was applied to interpret and visualize the immunoenrichment analysis terms for DEGs.

### 2.5 Identification of immune‐related DEGs associated with PTE

To further explore the expression profiling change of immune‐related DEGs caused by PTE, we first accessed immPort database (https://www.immport.org/),^20^ and 1794 immune‐related genes were obtained. In addition then, the Venn diagram was conducted to screen out the overlapped genes through the online software: jvenn (http://www.bioinformatics.com.cn/),^21^ which were defined as immune‐related DEGs.

### 2.6 PPI network analysis of immune‐related DEGs and hub gene screening

To explore the physical and functional relationship between proteins encoded by immune‐related DEGs, the search tool for the retrieval of interacting genes (STRING 10.5) (https://www.string‐db.org/)^22^ was applied to construct a PPI network, based on data from automated text mining, high‐throughput tests and co‐expression networks. Then, a Cytoscape plug‐in, Cytohubba was used for identifying hub genes. It provided 11 algorithms to evaluate the importance of genes in the network, including degree, edge percolated component, maximum neighbourhood component, density of maximum neighbourhood component, maximal clique centrality (MCC) and six centralities (bottleneck, eccentricity, closeness, radiality, betweenness and stress) based on shortest path, among which MCC algorithm with high prediction accuracy is adopted to screen for hub genes.^23^

### 2.7 Molecular docking for representative reproductive tobacco toxicants and proteins encoded by hub genes

For docking studies, the two‐dimensional structures of representative reproductive tobacco toxicants were downloaded from Pubchem database (https://pubchem.ncbi.nlm.nih.gov/),^24^ and the structure of proteins encoded by hub genes was downloaded from the research collaboratory for structural bioinformatics protein data bank (RCSB PDB) (http://www.rcsb.org/).^25^ Subsequently, these molecular were dehydrated and hydrogenated. Then, AutoDock Tools‐1.5.6 software was used for preparing the protein structures, determining the target regions for ligand binding and analysing the docking results. In addition, the binding energy of the medicinal ingredients and the targets was compared with that of the original ligand. Finally, the ligand–receptor with the lowest binding energy was visualized with the PyMOL Molecular Graphics System (Version 2.2 Schrödinger, LLC).

### 2.8 Tissue localization, physicochemical properties of hub immune‐related DEGs

First, the tissue localization of proteins encoded by hub genes was obtained through the bioGPS (building your own mash‐up of gene annotations and expression profiles) website (http://biogps.org)^26^ and the Human Protein Atlas.^27^ In addition, to further perceive the physicochemical properties and the functions of the proteins encoded by the hub genes, we searched the database of ProtParam (https://web.expasy.org/protparam/),^28^ bioGPS, Genecards (https://www.genecards.org/)^29^ and Uniprot (https://www.uniprot.org/).^30^

### 2.9 Consensus clustering analysis based on the immune‐related DEGs in PTE placenta

Considering that different immune landscapes existed in different PTE populations, we utilized unsupervised cluster analysis to identify different subtypes with the ‘ConsensusClusterPlus package’ in R software based on the gene expression profiles of the immune‐related DEGs in PTE placenta. In this process, the maximum number of clusters for the consistency clustering analysis was 9, replicates were 50, with a sample share of 0.8 and a feature‐to‐sample ratio of 1. To ensure the robustness of the classification, 1000 iterations were performed. In addition, then, the partitioning around medoids (PAM) clustering algorithm was selected to perform the clustering analysis with a euclidean distance function. Besides, cumulative distribution function (CDF) curve was drawn, where the number of possible clusters in the dataset was obtained using the consensus clustering method through the Consensus Cluster Plus package in R language. The smooth curve represents a reasonable clustering result. After the clustering analysis, PCA was performed using the ‘ggplot2’ package, and the classification points were plotted to verify the clustering results.

### 2.10 Immune infiltration landscape analysis of distinct subtypes

In this study, CIBERSORT algorithm was utilized to evaluate 22 immunocyte profiling in distinct subtypes obtained by consensus clustering analysis with ‘CIBERSORT’ package.^31^ Differences of immunocyte composition in subtypes were presented as heat maps and box plots. Besides, the expression profiling of immune checkpoints and human leukocyte antigen (HLA) gene sets were quantified by Kruskal–Wallis test. Furthermore, to screen out genes that contribute to the differential immuno‐infiltration characteristics of subtypes, Spearman correlation analysis was applied to explore the association between hub gens and the differentially infiltrated immunocytes in distinct subtypes by using the ‘corrplot’ package in R software. *P* < 0.05 was considered statistically significant.

### 2.11 Construction of immunocyte communication network

To explore how immune‐related DEGs are involved in the communication between differentially immunoinfiltrated cells, we constructed an immunocyte communication network. The cells in the immunocyte communication network were composed of differentially infiltrated immunocytes in different subgroups obtained by immune infiltration landscape analysis. The ligands and receptors that connect cells are obtained as follows. First, we acquired ligand–receptor pairing information from curated ligand–receptor pairs database and obtained average cap analysis of gene expression (CAGE) data for all ligands and receptors in the 144 human primary cells from the previously published study.^32^ Next, the immune‐related DEGs were intersected with the curated ligand–receptor pairs, and immunocytes corresponding to the overlapped genes were identified. Finally, the immunocyte communication network interfaced by immune‐related DEGs as ligands/receptors was visualized by Cytoscape 3.7.1 software.

### 2.12 Gene co‐expression modules with regard to differentially infiltrating immunocytes by WGCNA

The WGCNA is a widely used method to uncover critical interacted genetic modules and key genes by linking gene networks to clinical traits.^33^ In order to get the module genes that were closely associated with differentially infiltrating immunocytes, the co‐expression system was established on DEGs from GSE7434 and GSE27272 datasets by using the ‘WGCNA’ package in R software, with differently infiltrated immunocytes as clinical features derived from immune infiltration analysis. First, check expression profile distribution of all samples and detect abnormal samples. After that, the appropriate soft threshold was determined by using the pickSoftThreshold function and validated by the correlation between *k* and *p* (*k*). Subsequently, the correlation matrix was converted into an adjacency matrix, which was further processed into a topological overlap matrix (TOM). The dynamic tree cutting approach was performed to identify various modules. Finally, modules with the greatest spearman correlation coefficient were picked for further investigation.

### 2.13 Gene ontology and pathway enrichment analysis of the key modules

The genes in the key modules derived from WGCNA that were associated with PTE were imported into the Webgestalt website (http://www.webgestalt.org/)^34^ for Gene ontology biological process (GO‐BP) and the Kyoto Encyclopedia of Genes and Genomes (KEGG) pathway enrichment analysis. GO is a structured way to represent biological functions in terms of core entities and annotate protein biomarkers in the biological process (BP), cellular composition (CC) and molecular function (MF) levels.^35^ KEGG enables the correlation of gene catalogues to system functions at the cellular, species and ecosystem levels, facilitating researchers to understand the signalling pathways in which genes are involved.^36^ In the Webgestalt website, the species were selected as ‘Homo sapiens’, and the reference was set as genome protein‐coding. Items with a *p* value <0.05 were displayed in a circle plot by the ‘GOplot’ package in R software.

### 2.14 Determining signature genes for predicting placental immune disorders due to PTE by machine learning

Secreted proteins are potential biomarkers for pathologic diagnosis. In this part, we retrieved the Human Protein Atlas website (https://www.proteinatlas.org/) to download the dataset of secreted protein‐encoding genes. In addition, then, the overlapped genes of secreted protein‐encoding genes and immune‐related DEGs were obtained by using the Venn diagram tool, which were taken as potential biomarkers of immune disorders due to PTE, namely secretory protein‐encoding DEGs. Subsequently, machine learning methods were utilized to screen signature genes base on them.

First, one‐way logistic regression analysis was performed to find genes closely associated with PTE among the secretory protein‐encoding DEGs. Afterwards, the random forest (RF) algorithm was employed to further screen signature genes based on the result of one‐way logistic regression, bounded by MeanDecreaseGini greater than 0.8 for variable filtering. Similarly, the least absolute shrinkage and selection operator (LASSO) regression was also utilized based on critical genes recognized from the one‐way logistic regression analysis. After that, Venn diagrams were constructed to obtain the overlapped signature genes of RF and LASSO. In addition, we constructed an artificial neural network (ANN) model for the signature genes obtained from the above methods according to the gene score by using package of ‘neuralnet’ and ‘neuralnettools’ in R software. In addition, ROC was utilized to evaluate the accuracy of the ANN model in the training set (GSE7434 and placenta samples of GSE27272) and the testing set (peripheral blood samples of GSE27272). Most importantly, the expression profiling of the overlapped signature genes were further validated in the peripheral blood samples included in the GSE27272, and the receiver operating characteristic (ROC) curve was constructed by ‘pROC’ package in R software to evaluate the prediction accuracy of these signature genes. Finally, ‘rms’ package in R software was also utilized to establish the nomogram prediction model. Each signature gene was converted into an assessment point system in the model. The total score determined the final risk assessment value. Besides, the performance of the nomogram was assessed by calibration and discrimination,^37^ which was, respectively, evaluated by a visual calibration plot and the ROC. The visual calibration plot and the receiver operating characteristic curve were, respectively, constructed by the ‘rms’ and ‘pROC’ packages in R software, and the area under the ROC (AUC) was calculated. The closer the apparent line was to the ideal line and the closer the AUC was to 1, the better the prediction performance of the model was.^38^

### 2.15 Screening potential drugs for placental immune disorders due to PTE

Potential compounds that were able to target hub genes and ameliorate immune disorders due to PTE were predicted via the following database, including Comparative Toxicogenomics Database (CTD) (http://ctdbase.org/),^39^ the Drugbank (https://go.drugbank.com/)^40^ and the drug–gene interaction database (DGIdb, www.dgidb.org/).^41^ Drugs that meet the following conditions were included: (1) drug‐gene pairs with interaction score ≥0.1; (2) drugs supported by experiment, under clinical investigation or approved by the FDA. In addition, the correspondence between the drug and the target was visualized by Cytoscape software.

### 2.16 PTE mouse model to explore the effect of Nicotine on CCL18 and IFNA4 and the regulation of cyclosporine (CsA)

#### 2.16.1 Animals and administration schedule

Fifteen males and thirty females SPF C57BL/6 mice were provided by Shanghai SLAC animal Co., LTD. The production licence number of the experimental animal was SCXK (Zhejiang) 2017‐0005. The animals were raised in the Laboratory Animal Center (LARC) of Zhejiang Chinese Medical University, and the licence number of the Laboratory animal is SYXK (Zhejiang) 2021‐0012. 6–8 weeks of age, the body weight of mice was 20 ± 2 g, respectively. The ambient temperature must fluctuate within 22 ± 1°C, and the humidity must fluctuate between 50% and 60%. Light (12 h day/12 h night). Female mice were randomly divided into control group, nicotine exposure group and nicotine exposure+CsA group. Female and male rats closed their cages at 2:1 and began to be administered on the first day of discovery of the vaginal suppository. The control group was injected with sterile 0.9% physiological saline. Nicotine hydrogen tartrate salt was purchased from Aladdin (Cat. 6019‐06‐3, Aladdin) dissolved in sterile 0.9% physiological saline and adjusted to pH 7.2–7.4 with sodium hydroxide.^42^ Nicotine exposure group: Nicotine was administered subcutaneously in a volume of 0.1 mL, and the concentration of nicotine (3 mg/kg) was determined in previous studies.^43^ Nicotine exposure +CsA group: given CsA (Cat. C106893, Aladdin) intraperitoneal injection (25 mg/kg), every other day. The administration time of the three groups was 13 days, and then, the mice were anaesthetised and killed to obtain peripheral blood and placenta tissue for subsequent experiments. This study was approved by the Laboratory Animal Center of Zhejiang Chinese Medical University (Ethics number: 20190909‐07).

### 2.17 Enzyme‐linked immunosorbent assay (ELISA) for detection of serum CCL18 and IFNA4 levels

The serum samples were taken from the whole blood of mice without anticoagulant and centrifuged at 4°C and 3000 rpm for 15 min, and the supernatant was taken. Use ELISA kit to determine serum CCL18 (YX‐E20075, Shanghai Yuanxin Biotechnology) and IFNA4 (YX‐E21896, Shanghai Yuanxin Biotechnology) levels according to the corresponding instructions.

### 2.18 Detection of CCL18 and IFNA4 protein expression in placenta by Western blot

The RIPA buffer (Cat. P0013B, Beyotime biotechnology), protease inhibitors (Cat. No. B14002; Bimake), phosphatase inhibitors (Cat. B15002, Bimake) and pierceTM universal nuclease for placenta sample lysis (Cat. 88700, Thermo Scientific). First, gel electrophoresis was performed, and the concentrated glue voltage was adjusted to 80v and the separated glue voltage to 100v. The protein was then transferred to the Nitrocellulose (NC) film using a Mini TransBLot® electrophoresis transfer tank (Bio Rad Laboratories GmbH). The condition was set to 300 mA or 70 min. After transmembrane, the film was closed for 1 h, and primary antibodies (Cat. Abs149313, CCL18, Absin, 1:1000; Cat. Bs‐6304R, IFNA4, Bioss, 1:1000; Cat. 5174, GAPDH, CST, 1:1000) were incubated overnight at 4°C. The next day, goat anti‐rabbit IgG (H&L) was added (Cat. 926–32,211, LI‐COR, 1:5000). After the film was scanned in the Odyssey fluorescence imaging system, quantitative analysis was performed by Image J software (National Institutes of Health, Bethesda, Maryland).

### 2.19 Detection of CCL18 and IFNA4 protein expression in placenta by immunofluorescence

The immunofluorescence method was used to detect the content of CCL18 and IFNA4 in placenta. Frozen sections of the placenta tissue of mice were taken, washed and blocked, and then, CCL18 and IFNA4 antibodies (1:200) were added and incubated overnight at 4°C. Then, the samples were incubated with goat anti‐rabbit IgG H&L (Cy3®) in the dark (1:200) for 2 h. After that, the antifade mounting medium with DAPI was added. The CCL18 and IFNA4 were discovered by digital pathological section (fluorescence) scanning analyser (VS120‐S6‐W, OLYMPUS) and were analysed by Image J software. Besides, three sections and three different fields of each section were randomly selected for semi‐quantitative evaluation.

### 2.20 Calculation of embryo absorption rate

The embryo absorption rate is calculated by the following formula: Embryo absorption rate = number of absorbed foetuses/(number of absorbed foetuses + number of surviving foetuses) × 100%.

### 2.21 Statistical analysis

All results were expressed as Mean ± SEM. One‐way anova was used to analyse ELISA, Western blotting and immunofluorescence, followed by Dunnett's test for the post hoc test. In all calculations, a difference of *p* < 0.05 was considered significant. All data were plotted in GraphPad Prism 8 (GraphPad Software, San Diego, CA, United States).

## 3 RESULTS

### 3.1 GSEA associated with PTE

GSEA method was applied to appraise the differences in activations of KEGG pathway between the PTE placenta and health controls. Our results revealed that a total of 31 pathways were obtained (*p* < 0.05), and the results of the top 10 pathways sorted according to the normalized enrichment scale were shown in Figure S2. Among them, several intrinsic and adaptive immunity‐related pathways were activated in PTE samples including natural killer (NK) cell mediated cytotoxicity, T‐cell receptor signalling pathway and Th1/Th2 cell differentiation, etc.

### 3.2 Identification of DEGs and immune enrichment analysis of DEGs

**FIGURE 2:**
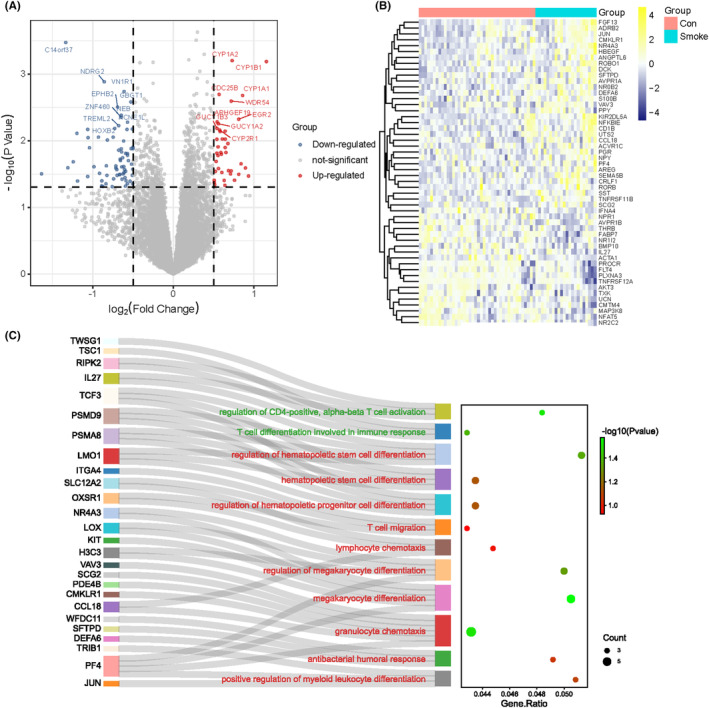
Differentially expressed gene analysis and immunoenrichment analysis. (A) Volcano plot and (B) Heat map visualized the DEGs. In the volcano plot, each dot represents a gene. Red plot points represent up‐regulated genes, and blue plot points represent down‐regulated, and in the heat map, each row represents a DEG, and each column represents a sample. (C) Immune enrichment analysis on up‐regulated and down‐regulated DEGs using ClueGO.

After preprocessing and normalization of the included datasets, we performed differential expression analysis. Totally, 680 DEGs were obtained, among which 374 were up‐regulated, and 306 genes were down‐regulated (*p* < 0.05) (Figure 2A,B). To investigate the potential of DEGs in immune regulation, we performed immune enrichment analysis on up‐regulated and down‐regulated DEGs using ClueGO, respectively. The results showed that the up‐regulated DEGs were mainly enriched in regulation of haematopoietic stem cell differentiation, T‐cell migration and megakaryocyte differentiation, etc., and the down‐regulated DEGs were mainly enriched in T‐cell differentiation involved in immune response and regulation of CD4‐positive alpha‐beta T‐cell activation, etc (Figure 2C). It suggested that PTE contributed to extensive differential gene expression that were closely related to immune process of the placenta.

### 3.3 Identification of immune‐related genes associated with PTE

**FIGURE 3:**
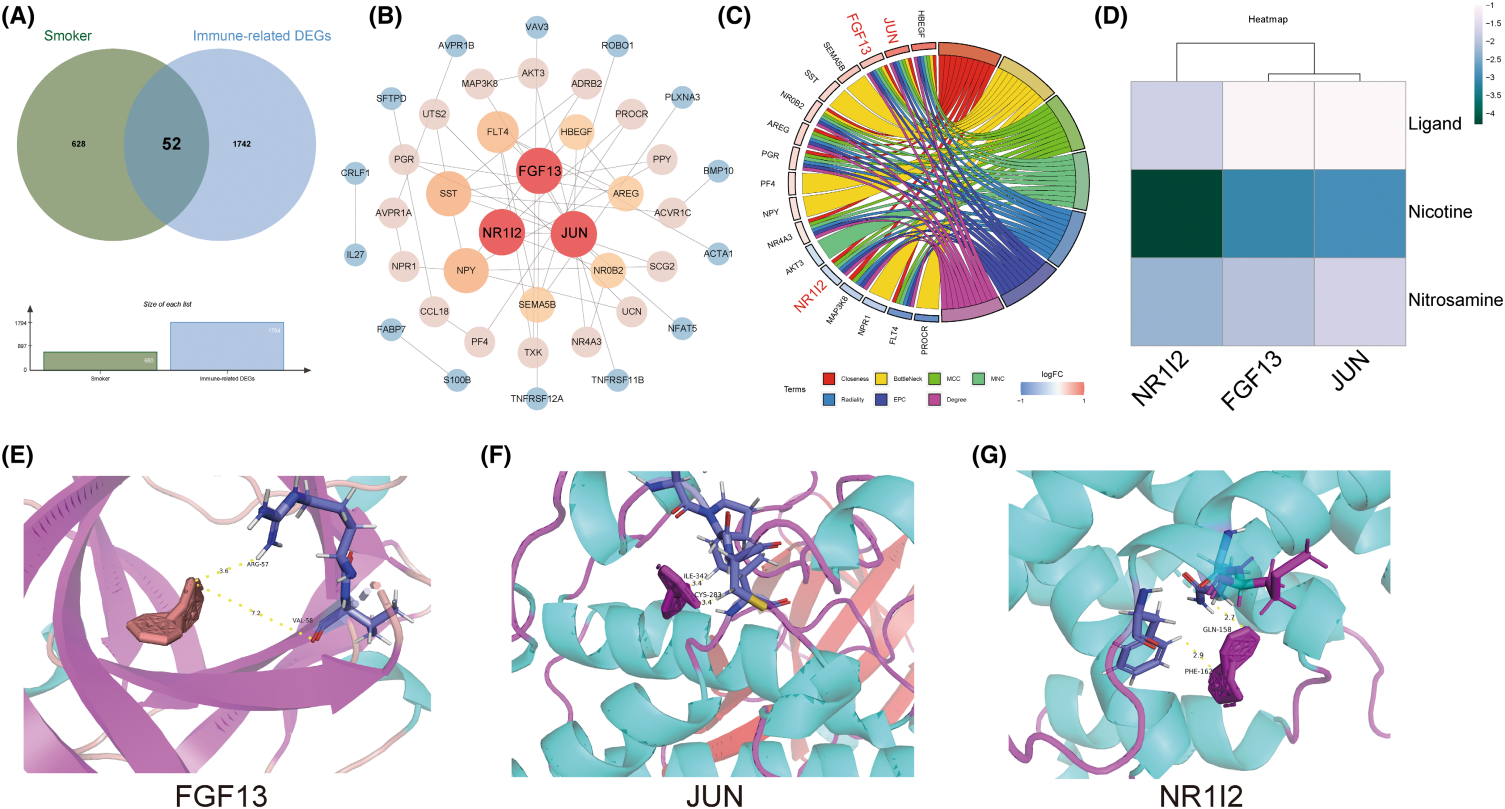
Identification and PPI network analysis of immune‐related genes and molecular docking of representative reproductive toxicants and proteins encoded by hub genes. (A) Identification of immune‐related DEGs by venn plot. (B) Protein–protein interaction network analysis of immune‐related DEGs. Nodes from large to small, the colour from red to blue represents the degree value from large to small. (C) The chord diagram shows the top 10 hub genes under the MCC algorithm. (D) The heatmap showed the molecular docking binding energy of two representative reproductive toxins in cigarette in cigarette with its own ligands and JUN, FGF13, NR1I2. The colour from white to dark green represents the binding energy from high to low. (E–G) The molecular docking patterns of FGF13, JUN, NR1I2 and nicotine are shown respectively.

Given that DEGs were involved in a variety of immune regulation processes, we first obtained 1794 immune‐related gene from the ImmPort database, and then intersected them with the DEGs in PTE placenta. Finally, we obtained a total of 52 overlapping genes, which were taken as a new gene set for subsequent analysis, namely immune‐related DEGs (Figure 3A).

### 3.4 PPI network analysis of immune‐related DEGs and identification of hub genes

To unravel the protein interactions and to screen DEGs that were strongly linked to PTE, we performed PPI network analysis on the basis of the 52 immune‐related genes using the STRING database and visualized the network with Cytoscape software, as shown in Figure 3B. The top 10 hub genes under the MCC algorithm included *JUN, NPY, SST, FLT4, FGF13, HBEGF, NR0B2, AREG, NR1I2, SEMA5B*, among which 3 hub gene, *JUN, FGF13* and *NR1I2*, were ranked in the top 10 in all the seven algorithms in Cytohubba (Figure 3C, Table 2).

**TABLE 2:** JUN, FGF13 and NR1I2 were ranked in the top ten in all the seven algorithms in Cytohubba.

Closeness	BottleNeck	MCC	MNC	Radiality	EPC	Degree
JUN	JUN	JUN	NPY	JUN	JUN	JUN
FGF13	FGF13	NPY	SST	FGF13	FGF13	NPY
FLT4	NPY	SST	JUN	HBEGF	HBEGF	SST
HBEGF	FLT4	FLT4	NR0B2	FLT4	NR0B2	FLT4
NR1I2	SST	FGF13	FGF13	NR1I2	FLT4	FGF13
NR0B2	NR1I2	NR0B2	NR1I2	PGR	AREG	HBEGF
PGR	PROCR	NR1I2	HBEGF	NR0B2	NR1I2	NR0B2
AREG	SEMA5B	SEMA5B	NR4A3	AREG	NR4A3	AREG
NR4A3	PF4	AREG	MAP3K8	NR4A3	PGR	NR1I2
MAP3K8	NPR1	HBEGF	AKT3	MAP3K8	MAP3K8	SEMA5B

### 3.5 Molecular docking of representative reproductive toxicants and proteins encoded by hub genes

Here, we selected two representative reproductive toxicants in cigarette, nicotine and nitrosamine, for molecular docking with proteins encoded by representative hub genes, *JUN, FGF13* and *NR1I2*. The results demonstrated that the binding energy of JUN, FGF13 and NR1I2 to nicotine and nitrosamine was lower than that of their own ligands, with the binding energy of JUN, FGF13 and NR1I2 to Nicotine being the lowest (−3.07, −2.91 and −4.32, respectively), as shown in Figure 3D–G. This indicated that nicotine and nitrosamine could bind to JUN, FGF13 and NR1I2 with high affinity, which might act as vital anchors for the pathological signal transduction in placenta triggered by PTE.

### 3.6 Tissue localization and physicochemical properties of hub immune‐related DEGs

According to the bioGPS and the Human Protein Atlas websites, the hub genes were expressed in placenta, uterus as well as multiple immunocytes. For example, *JUN* was expressed in CD19+ B cells, BDCA4+ dendritic cells, CD33+ myeloid cells and CD14+ monocytes. *FLT4* was expressed in CD8+ T cells. *HBEGF* was expressed in CD19+ B cells, CD4+ T cells, CD56+ NK cells and CD14+ monocytes. Localization on immunocytes is an essential prerequisite for the above genes to be able to regulate placental immune function (Table 3). The physicochemical properties of the reported hub proteins are showed in Table 4.

**TABLE 3:** The function, cell localization and 3D structure of the reported hub genes.

Gene Name	Description	Function	Cell localization	3D Structure
JUN	Jun Proto‐Oncogene, AP‐1 Transcription Factor Subunit	Playing a role in activation‐induced cell death of T cells by binding to the AP‐1 promoter site of FASLG/CD95L, and inducing its transcription in response to activation of the TCR/CD3 signalling pathway (PubMed: 12618758).	Uterus, CD19+ B cells, BDCA4+ Dendritic cells, CD33+ myeloid, CD14+ monocytes	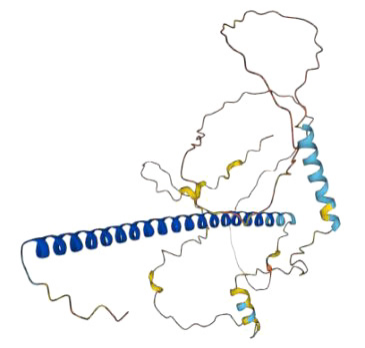
NPY	Neuropeptide Y	This gene encodes a neuropeptide that inhibits adenylate cyclase through G protein‐coupled receptors, activates mitogen‐activated protein kinase (MAPK), regulates intracellular calcium levels and activates potassium channels. The protein also exhibits antibacterial activity against bacteria and fungi.	Foetal brain	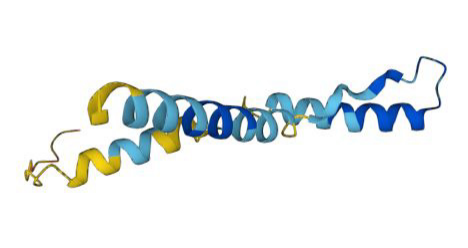
SST	Somatostatin	May enhance low‐glucose‐induced glucagon release by pancreatic alpha cells (By similarity). This effect may be mediated by binding to GPR107 and PKA activation (By similarity). May regulate cardiac contractile function (By similarity). May compromise cardiomyocyte viability (By similarity). In the central nervous system, may impair memory retention and may affect hippocampal excitability (By similarity). May also have anxiolytic and anorexigenic effects (By similarity). May play a role in arterial pressure regulation (By similarity). May inhibit basal, but not ghrelin‐ or GnRH‐stimulated secretion of GH1 or LH, but does not affect the release of other pituitary hormones, including PRL, ACTH, FSH or TSH. Potentiates inhibitory action of somatostatin on ghrelin‐stimulated secretion of GH1, but not that on GnRH‐stimulated secretion of LH (PubMed: 29615476).	–	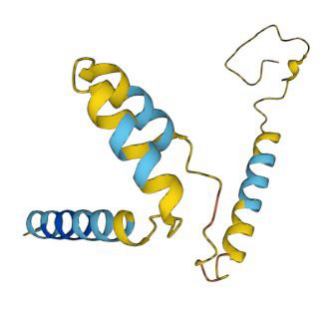
FLT4	Fms Related Receptor Tyrosine Kinase 4	Tyrosine‐protein kinase that acts as a cell‐surface receptor for VEGFC and VEGFD, and plays an essential role in adult lymphangiogenesis and in the development of the vascular network and the cardiovascular system during embryonic development. Promotes proliferation, survival and migration of endothelial cells, and regulates angiogenic sprouting. Signalling by activated FLT4 leads to enhanced production of VEGFC, and to a lesser degree VEGFA, thereby creating a positive feedback loop that enhances FLT4 signalling. Modulates KDR signalling by forming heterodimers. The secreted isoform 3 may function as a decoy receptor for VEGFC and/or VEGFD and play an important role as a negative regulator of VEGFC‐mediated lymphangiogenesis and angiogenesis. Binding of vascular growth factors to isoform 1 or isoform 2 leads to the activation of several signalling cascades; isoform 2 seems to be less efficient in signal transduction, because it has a truncated C‐terminus and therefore lacks several phosphorylation sites.	Placenta, CD8+ T cells	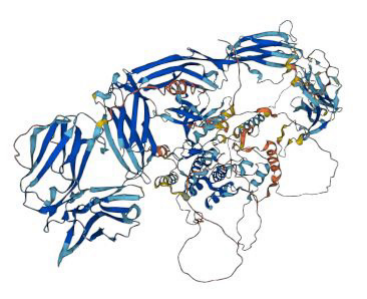
FGF13	Fibroblast Growth Factor 13	The protein encoded by this gene is a member of the fibroblast growth factor (FGF) family, which has a wide range of mitogenic and cell survival activities and is involved in a variety of biological processes, including embryonic development, cell growth, morphogenesis, tissue repair, tumour growth and invasion.	Foetal brain	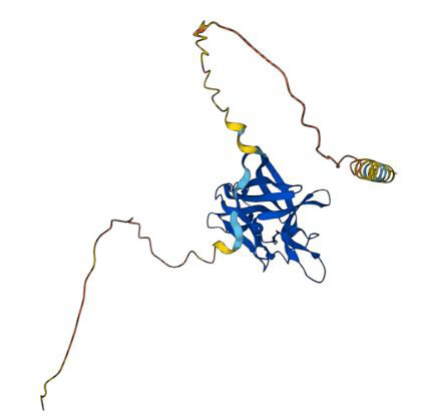
HBEGF	Heparin Binding EGF Like Growth Factor	Growth factor that mediates its effects via EGFR, ERBB2 and ERBB4. Required for normal cardiac valve formation and normal heart function. Promotes smooth muscle cell proliferation. May be involved in macrophage‐mediated cellular proliferation. It is mitogenic for fibroblasts, but not endothelial cells. It is able to bind EGF receptor/EGFR with higher affinity than EGF itself and is a far more potent mitogen for smooth muscle cells than EGF. Also acts as a diphtheria toxin receptor.	B lymphoblasts, CD19+ B cells, BDCA4+ Dendritic cells, CD4+ T cells, CD56+ NK cells, CD33+ myeloid, CD14+ monocytes	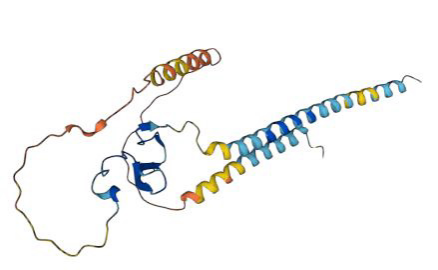
NR0B2	Nuclear Receptor Subfamily 0 Group B Member 2	Transcriptional regulator that acts as a negative regulator of receptor‐dependent signalling pathways (By similarity). Specifically inhibits transactivation of the nuclear receptor with which it interacts (By similarity). Inhibits transcriptional activity of NEUROD1 on E‐box‐containing promoter by interfering with the coactivation function of the p300/CBP‐mediated transcription complex for NEUROD1 (PubMed: 14752053). Essential component of the liver circadian clock which via its interaction with NR1D1 and RORG regulates NPAS2‐mediated hepatic lipid metabolism (By similarity). Regulates the circadian expression of cytochrome P450 (CYP) enzymes (By similarity). Represses: NR5A2 and HNF4A to down‐regulate CYP2C38, NFLI3 to up‐regulate CYP2A5, BHLHE41/HNF1A axis to up‐regulate CYP1A2, CYP2E1 and CYP3A11, and NR1D1 to up‐regulate CYP2B10, CYP4A10 and CYP4A14 (By similarity).	–	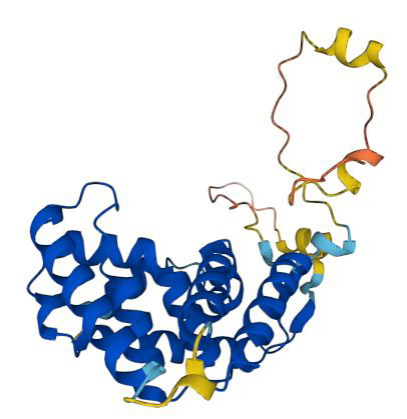
AREG	Amphiregulin	Ligand of the EGF receptor/EGFR. Autocrine growth factor as well as a mitogen for a broad range of target cells including astrocytes, Schwann cells and fibroblasts.	CD34+, B lymphoblasts, CD56+ NK cells, CD33+ myeloid, CD14+ monocytes	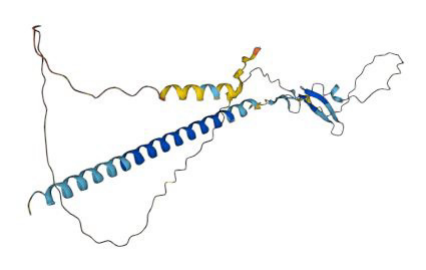
NR1I2	Nuclear Receptor Subfamily 1 Group I Member 2	It is a transcription factor characterized by ligand‐binding and DNA‐binding domains and belongs to the nuclear receptor superfamily, which binds and is activated by various endogenous and exogenous compounds, thus prompting transcription of multiple genes.	CD34+, B lymphoblasts, CD56+ NK cells, CD33+ myeloid	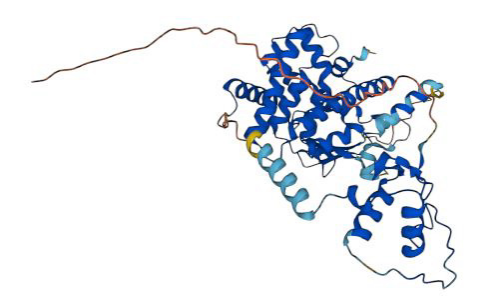
SEMA5B	Semaphorin 5B	This gene encodes a member of the semaphorin protein family which regulates axon growth during development of the nervous system. The encoded protein has a characteristic Sema domain near the N‐terminus, through which semaphorins bind to plexin, and five thrombospondin type 1 repeats in the C‐terminal region of the protein. The protein product may be cleaved and exist as a secreted molecule (PMID: 19463192). Multiple transcript variants encoding different isoforms have been found for this gene.	–	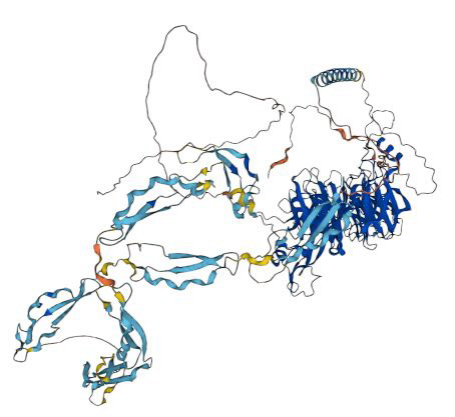

*Note*: Model confidence: Very high (pLDDT >90); Confident (90> pLDDT >70); Low (70> pLDDT >50); Very low (pLDDT <50). AlphaFold produces a per‐residue confidence score (pLDDT) between 0 and 100. Some regions below 50 pLDDT may be unstructured in isolation.

**TABLE 4:** The physicochemical properties of the reported hub proteins.

Name	Number of amino acids	Molecular weight (kda)	Theoretical pI	Number of Negatively charged residues (Asp + Glu)	Number of positively charged residues (Arg + Lys)	Extinction coefficient	Instability index	Aliphatic index	Grand average of hydropathicity (GRAVY)
JUN	331	35675.57	8.90	28	32	7575	53.28	74.65	−0.469
NPY	36	4272.72	6.76	5	5	7450	63.74	54.44	−1.194
SST	28	3150.60	9.85	1	5	5625	34.77	14.29	−0.732
FLT4	1339	150216.22	5.87	171	145	213,205	50.94	81.92	−0.349
FGF13	245	27563.58	9.92	20	42	23,630	58.47	67.63	−0.685
HBEGF	86	9729.32	9.34	11	19	4845	48.66	70.35	−0.909
NR0B2	257	28057.81	8.28	20	23	36,730	64.70	104.44	0.132
AREG	87	10117.55	9.61	11	22	3355	36.04	32.41	−1.538
NR1I2	434	49761.64	8.70	53	61	34,920	51.73	75.30	−0.395
SEMA5B	1151	125912.97	7.99	98	105	201,335	60.93	67.39	−0.356

*Note*: Extinction coefficients are in units of M^−1^ cm^−1^, at 280 nm measured in water.

### 3.7 Immune infiltration landscape of distinct subtypes

**FIGURE 4:**
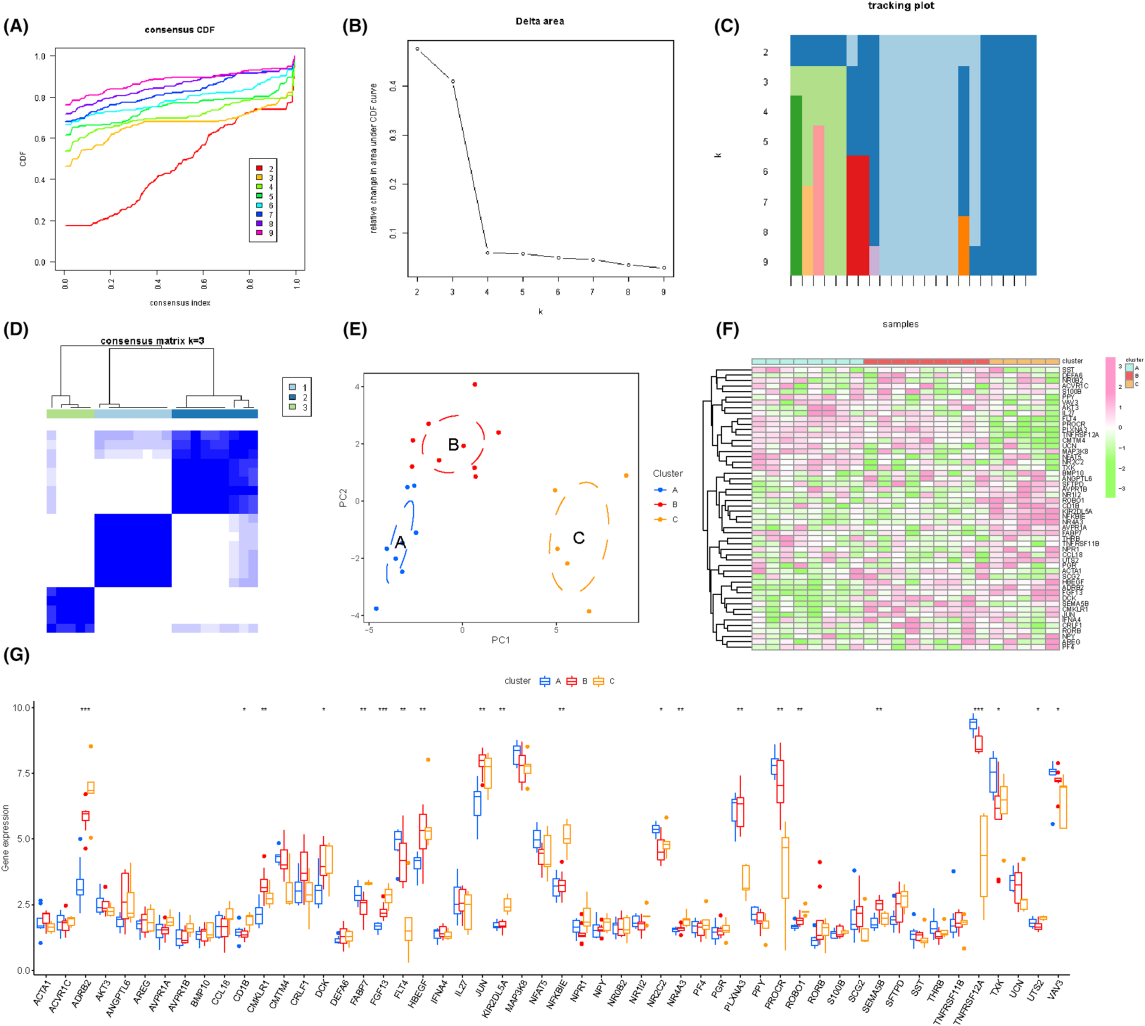
Consensus clustering analysis of the prenatal tobacco exposure samples. (A) The consensus clustering cumulative distribution function (CDF) for *k* = 2–9. (B) The relative change in area under the CDF curve for *k* = 2–9. (C) Item tracking plot showing the consensus cluster of items (in column) at each *k* (in row). (D) Consensus matrix plots depicting consensus values on a white to blue colour scale ordered by consensus clustering when *k* = 3. (E) t‐SNE plots confirming the classification accuracy of two distinct subtypes across the prenatal tobacco exposure samples. (F, G) Heatmap and box diagram showed the expression level of 52 immune‐related DEGs in the three subtypes. *represents *p* < 0.05, **represents *p* < 0.01, ***represents *p* < 0.001 among the three groups.

**FIGURE 5:**
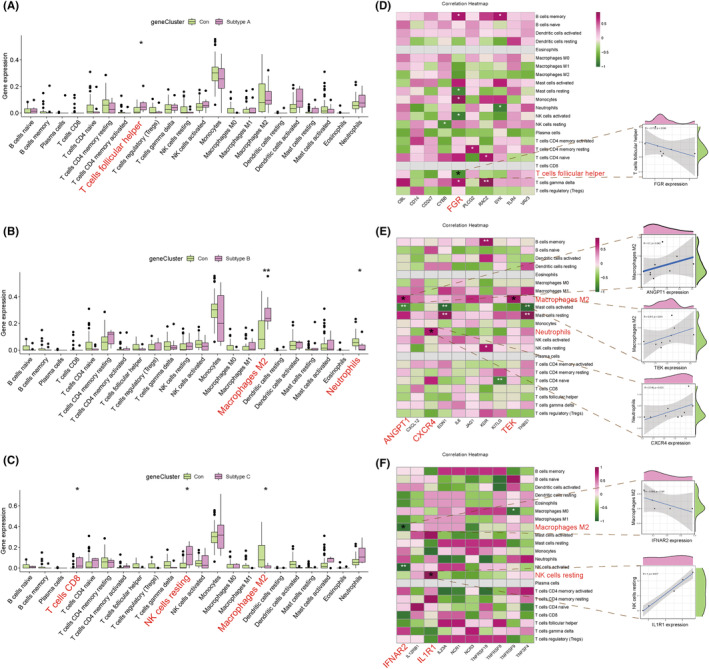
Immune infiltration landscape of distinct subtypes. (A–C) The box diagram showed the immunocyte infiltration of control group and subtype A/subtype B/subtype C group. (D–F) showed the genes closely associated with the immune infiltration characteristics in 3 subtypes by spearman correlation analysis. *represents *p* < 0.05 compared with the control group, **represents *p* < 0.01 compared with the control group.

By consensus clustering analysis, we clustered the samples into 3 subtypes, subtype A (*n* = 8), subtype B (*n* = 9) and subtype C (*n* = 5) based on the immune‐related DEGs in PTE placenta (Figure 4A–G). To uncover the immune infiltration landscape in subtypes, we used the CIBERSORT algorithm to quantify the level of immunocyte infiltration. The results revealed that when compared with non‐PTE samples, follicular T helper (fTh) cells were significantly elevated in subtype A (*p* < 0.05), M2 macrophages were elevated (*p* < 0.01) and neutrophils were significantly decreased (*p* < 0.05) in subtype B and CD8+ T cells, resting NK cells were significantly elevated (*p* < 0.05) and M2 macrophages were significantly decreased (*p* < 0.05) in subtype C (Figure 5A–C). The above results suggested that different expression patterns of immune‐related DEGs exerted a significant impact on the immune landscape feature due to PTE. Furthermore, we also assessed gene expression profile of the HLA related genes and immune checkpoints in subtypes, and the results were shown in Figure S3.

Besides, we also screened the genes closely associated with the immune infiltration characteristics in three subtypes by Spearman correlation analysis. The results displayed that in subtype A, *FGR* was significantly negatively correlated with fTh cells (*r* = −0.738, *p* < 0.05). In subtype B, *ANGPT1* (*r* = 0.700, *p* < 0.05) and *TEK* were positively associated with M2 macrophages (*r* = 0.817, *p* < 0.05), and *CXCR4* was positively associated with neutrophils (*r* = 0.746, *p* < 0.05). In subtype C, *IFNAR2* exhibited a significant negative correlation with M2 macrophages (*r* = −0.894, *p* < 0.05), and *IL1R1* showed a significant positive correlation with resting NK cells (*r* = 1.000, *p* < 0.05) (Figure 5D–F).

### 3.8 Construction of immunocyte communication network

**FIGURE 6:**
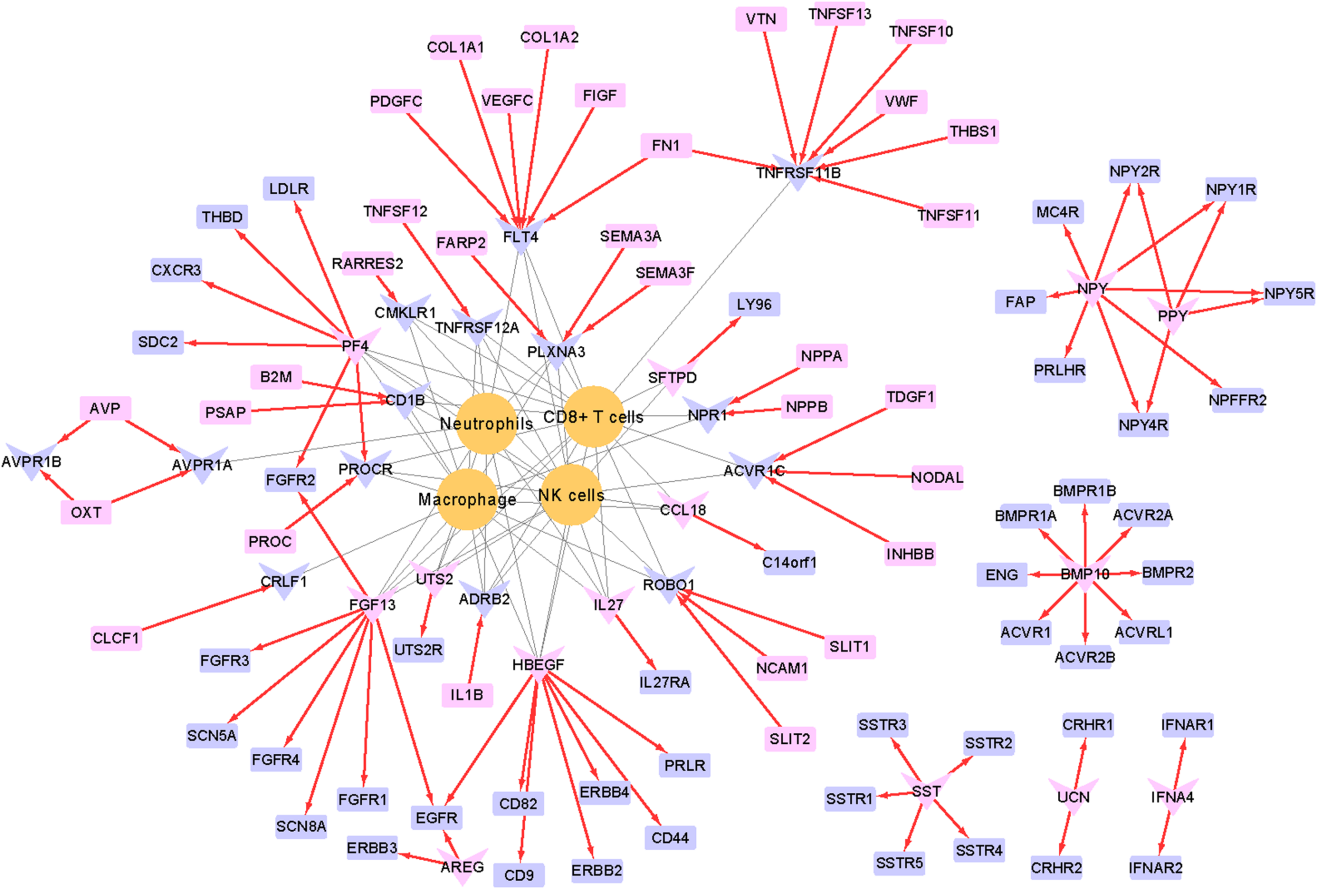
Construction of immunocyte communication network interfaced by ligand–receptors from immune‐related DEGs. In this figure, the orange circular node represents different immune infiltrating cells, the blue node represents receptor molecules, the pink node represents ligand molecules, the rectangular node represents genes from curated ligand–receptor pairs database, and the V‐type node represents immune‐related DEGs. Red arrow points from ligand to receptor.

In this part, 28 genes as ligands or receptors among the 52 immune‐related DEGs were obtained. Among them, 14 genes acted as ligands corresponding to 62 receptors, and 14 genes acted as receptors, corresponding to 50 receptors. In addition, 20 of the 28 genes that acted as ligands (or receptors) corresponded to CD8+ T cells, neutrophils, macrophage and NK cells derived from immune infiltration analysis in subtypes. Finally, Cytoscape was used to visualize the immunocyte communication network interfaced by the 20 ligands or receptors, as shown in Figure 6.

### 3.9 Gene co‐expression modules with regard to differentially infiltrating immunocytes by WGCNA

**FIGURE 7:**
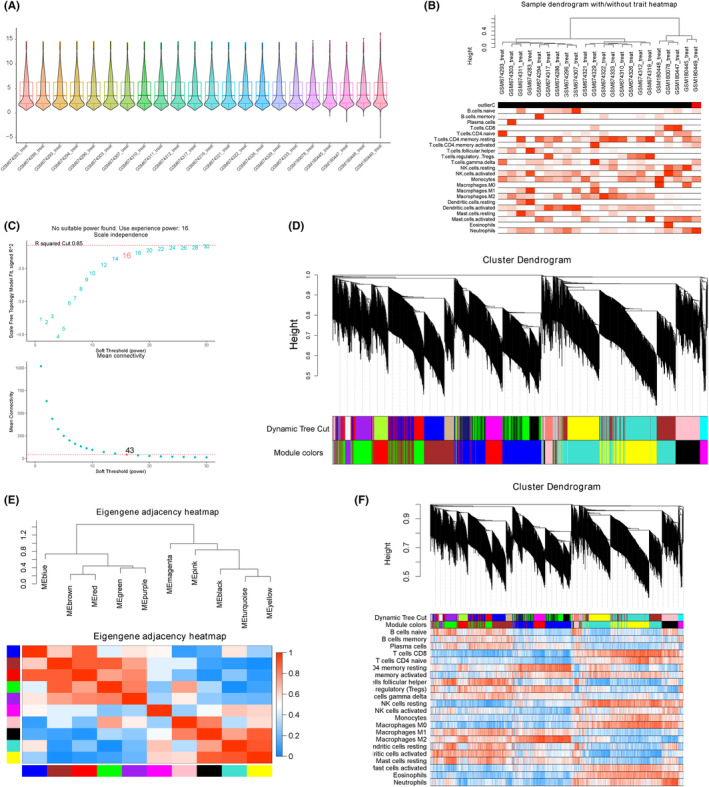
Gene co‐expression modules with regard to immune infiltration by WGCNA. (A) Boxplot plus violin plot showing the expression profile distribution of all samples. (B) Hierarchical clustering showing sample correlations and outlier samples. (Samples labelled with red bars in <outlierC> row are detected potential outlier samples). (C) Scale independence and mean connectivity under diverse soft‐thresholding powers. (D) WGCNA module plot. <Dynamic Tree Cut> represents initial modules. <Module colours> represent final modules. Each branch in the hierarchical tree or each vertical line in colour‐bars represents one gene. Genes not attributed to any module would be coloured by grey. (E) Correlation of all identified modules. Each colour represents one module. (F) WGCNA module gene trait correlation plot.

**FIGURE 8:**
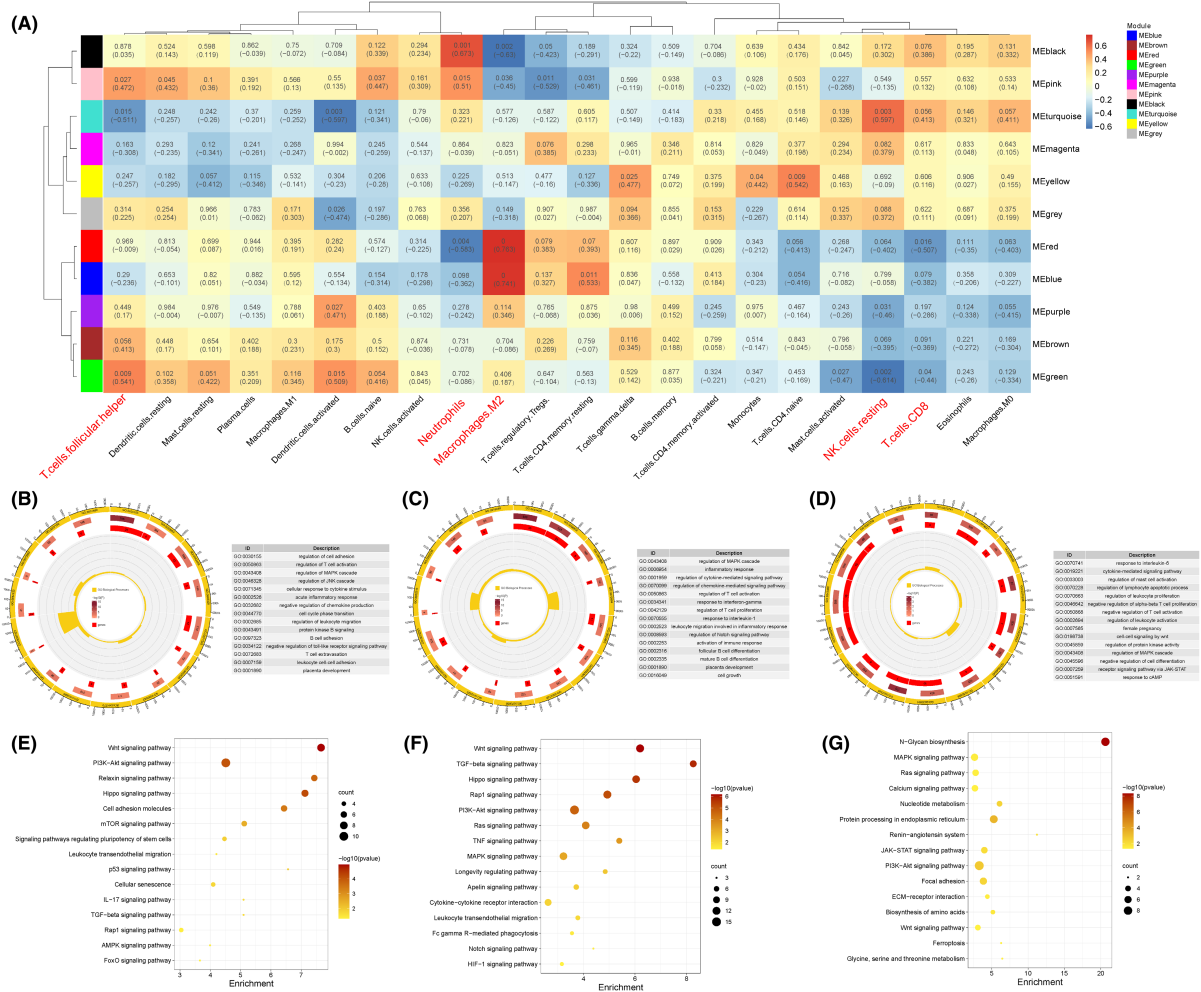
Gene ontology and pathway enrichment analysis for key modules. (A) WGCNA module trait correlation plot. Each row represents one module. Each column represents one trait attribute. Blue colour represents negative correlation, and red colour represents positive correlation. (B, E) Results of GO‐BP and KEGG enrichment analysis of red module gene. (C, F) Results of GO‐BP and KEGG enrichment analysis of blue module gene. (D, G) Results of GO‐BP and KEGG enrichment analysis of green module gene.

First, we examined the distribution of expression profiles in 22 PTE placental samples, all of which were included for WGCNA to identify gene co‐expression modules associated with immune cell infiltration. The soft power of *β* = 16 (scale‐free *R* ^2^ = 0.85) was determined as soft‐thresholding to acquire co‐expressed gene modules (Figure 7A–C). In addition, the module detection was performed by hierarchical clustering and dynamic tree cut functions (Figure 7D–F). Ultimately, a total of 10 modules were divided by WGCNA and were identified by different colours (Figure 8A). Among these gene modules, the red module was significantly correlated with neutrophils (*r* = −0.583, *p* = 0.004), M2 macrophages (*r* = 0.763, *p* = 0) and CD8+ T cells (*r* = −0.507, *p* = 0.016); the blue module was significantly correlated with M2 macrophages (*r* = 0.741, *p* = 0); the green module was significantly correlated with follicular helper T cells (*r* = 0.541, *p* = 0.009), resting NK cells (*r* = −0.614, *p* = 0.002), CD8+ T cells (*r* = −0.44, *p* = 0.04). As the genes in red, blue and green modules were positively or negatively relevant to the most immunocytes, they were considered as important transcriptionally dysregulated gene modules with regard to the altered immune microenvironment.

### 3.10 Gene ontology and pathway enrichment analysis of key modules

In addition, to further understand the biological pathways by which these genes impinge on the characteristics of immune infiltration, we performed GO and KEGG enrichment analysis on the gene in these three modules. The GO‐BP analysis revealed that genes in the red module were enriched in regulation of T‐cell activation, cellular response to cytokine stimulus and B cell adhesion (Figure 8B). Besides, genes in the blue module were mostly enriched in regulation of inflammatory response, regulation of T‐cell activation and mature B‐cell differentiation (Figure 8C). Genes in the green module were mostly enriched in cytokine‐mediated signalling pathway, regulation of lymphocyte apoptotic process and negative regulation of T‐cell activation (Figure 8D). KEGG results demonstrated that the red module genes were mostly enriched in PI3K‐Akt signalling pathway, leukocyte transendothelial migration and IL‐17 signalling pathway, etc (Figure 8E). The blue module genes were mostly enriched in TGF‐beta signalling pathway, TNF signalling pathway and cytokine‐cytokine receptor interaction, etc (Figure 8F). The green module genes were mostly enriched in MAPK signalling pathway, JAK–STAT signalling pathway and Wnt signalling pathway, etc (Figure 8G).

### 3.11 Determining signature genes for predicting immune disorders due to PTE by machine learning

**FIGURE 9:**
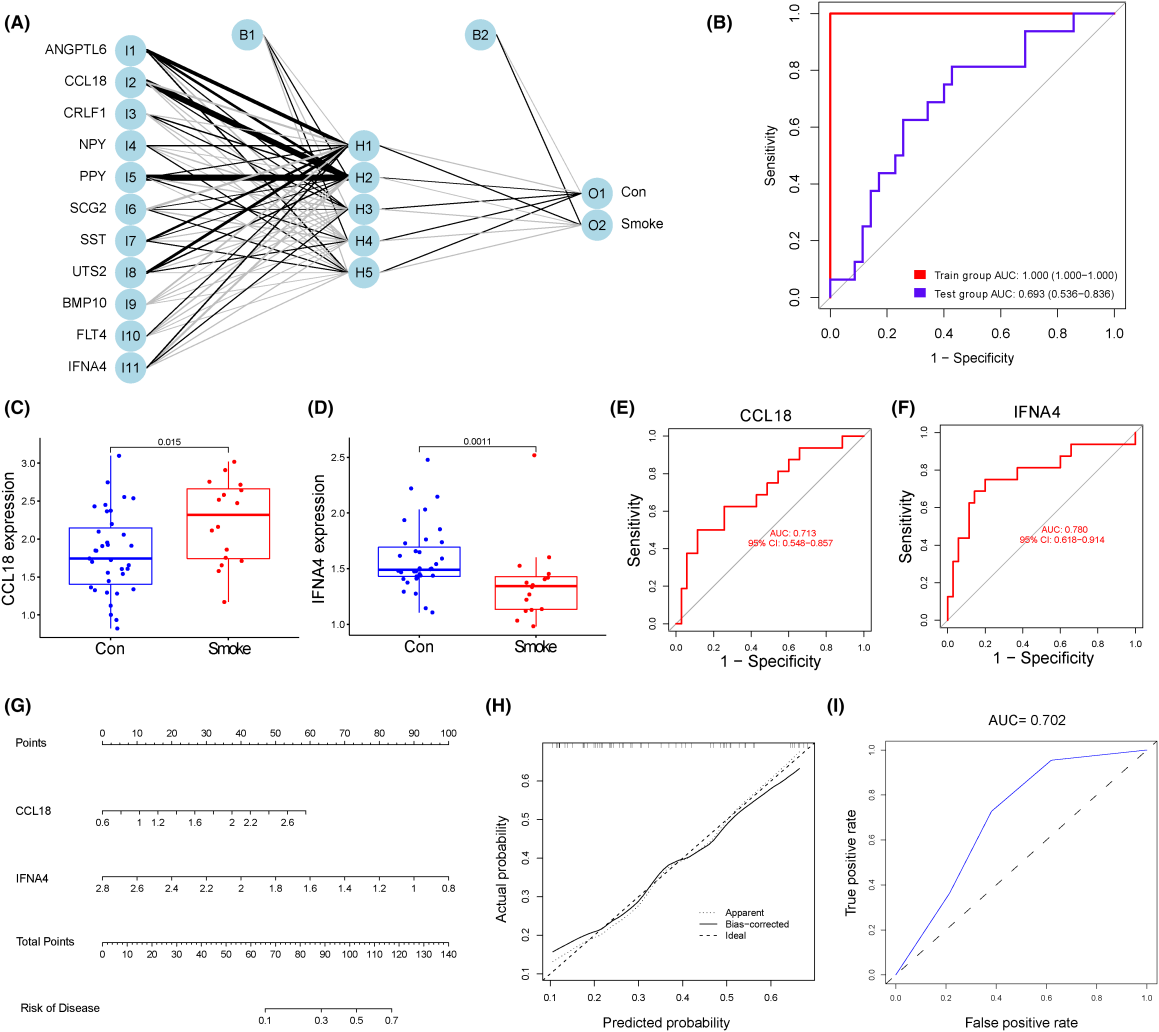
Determining signature genes for tobacco‐induced placental immune disorder by machine learning. (A) Neural network model: I1‐I11 is the input layer (8 signature genes score and weight), H1–H5 is the hidden layer, O1–O2 is the output layer (sample attributes). (B) ROC curves for evaluating the diagnostic efficacy of the neural network model in training set and testing set. (C, D) The mRNA expression profiling analysis of the CCL18 and IFNA4. (E, F) The ROC curves of CCL18 and IFNA4. (G) The nomogram prediction model. (H) The visual calibration plot for the internal validation of the prediction model. (I) ROC for assessing discriminative performance of the nomogram prediction model.

Given that secreted proteins are able to enter the peripheral circulation from tissues, they are potential to predict local histopathological alterations. Therefore, we screened biomarkers from immune‐related DEGs that could encode secreted proteins to predict placental immune abnormalities due to PTE. First, we took the intersection of immune‐related DEGs in placenta due to PTE with genes encoding secretory‐type proteins from the Human Protein Atlas website, and finally obtained 18 DEGs encoding secretory‐type proteins (Figure S4A). Subsequently, we took one‐way logistic regression analysis on the 18 genes, and the results revealed that 15 genes were strongly associated with PTE (Table S1). Next, RF was applied to screen out the genes from the 15 DEGs with MeanDecreaseGini greater than 0.8, and a total of 12 genes were obtained (Figure S4B,C). Besides, Lasso regression was performed on the 15 genes and obtained a total of 13 signature genes that could sensitively distinguish PTE placenta from normal controls (Figure S4D). Subsequently, 11 signature genes were obtained from the intersection of LASSO and RF algorithms (Figure S4E). In addition, these 11 genes were used for constructing neural network. Results showed that the 11 genes could distinguish the control samples from the PTE samples well, and the AUC of the training set and testing set was, respectively, 1.000 and 0.693, which demonstrated the high accuracy of the ANN model (Figure 9A,B). Among these genes, CCL18 was significantly increased (*p* = 0.015) and IFNA4 was significantly decreased (*p* = 0.0011) in peripheral blood in the PTE group (Figure 9C,D). The expression profiles of other genes between groups were shown in Figure S5A–I. Afterwards, ROC curves were constructed to determine the diagnostic value of CCL18 and IFNA4. The results indicated that the AUC of CCL18 and IFNA4 was 0.713 (0.548–0.857) and 0.780 (0.618–0.914), respectively, which identified the diagnostic value of CCL18 and IFNA4 (Figure 9E,F). The AUC of other genes was shown in Figure S5J–R. In addition, we constructed nomogram with the two genes (Figure 9G). In addition, the calibration curves verified the predictive performance of the model (Figure 9H), with high AUC value of 0.702 to verify its robusty (Figure 9I).

### 3.12 Screening potential drugs for placental immune disorders due to PTE

**FIGURE 10:**
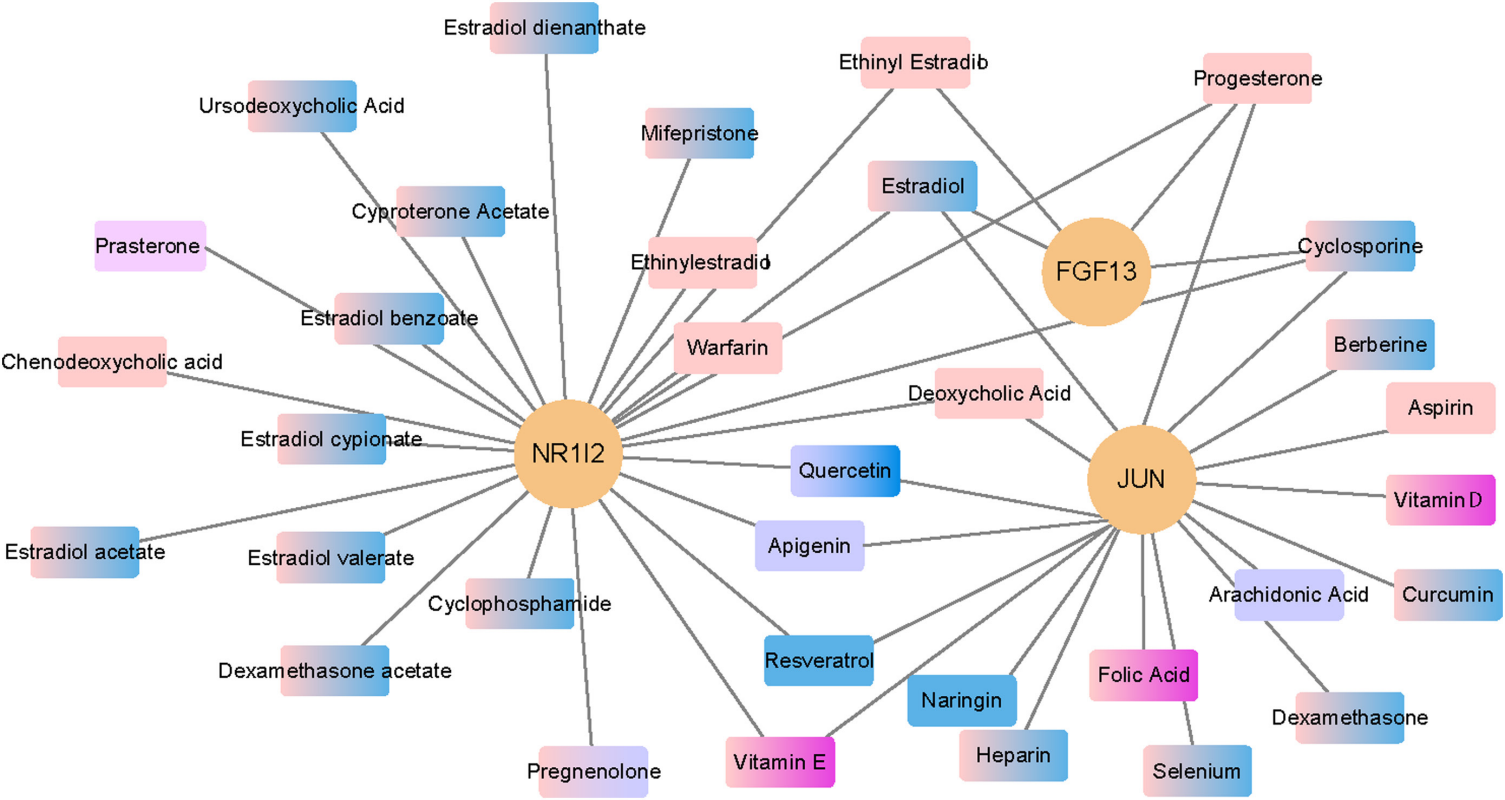
Potential compounds targeting hub genes. In this figure, the orange circular node represents hub genes, the apricot node represents approved drugs, the blue node represents investigational drugs, and the indigo node represents experimental drugs. The nodes of the blue‐apricot gradient represent approved and investigational drugs, the nodes of the apricot‐indigo gradient represent approved and experimental drugs, the nodes of the blue‐indigo gradient represent investigational and experimental drugs, the nodes of the apricot‐purple gradient represent approved and nutraceutical drugs, and the pink nodes represent approved, investigational and nutraceutical drugs.

A total of 34 compounds were predicted based on the representative hub genes as targets with CTD, the Drugbank and DGIdb databases. Oestradiol, cyclosporine, progesterone can act on *NR1I2, JUN* and *FGF13* at the same time (Figure 10). Besides, all the 34 compounds have been confirmed by experimental studies or approved by FDA. However, their protective effects in PTE need to be further confirmed.

### 3.13 Validation of signature genes and potential therapeutic drugs in PTE animals

**FIGURE 11:**
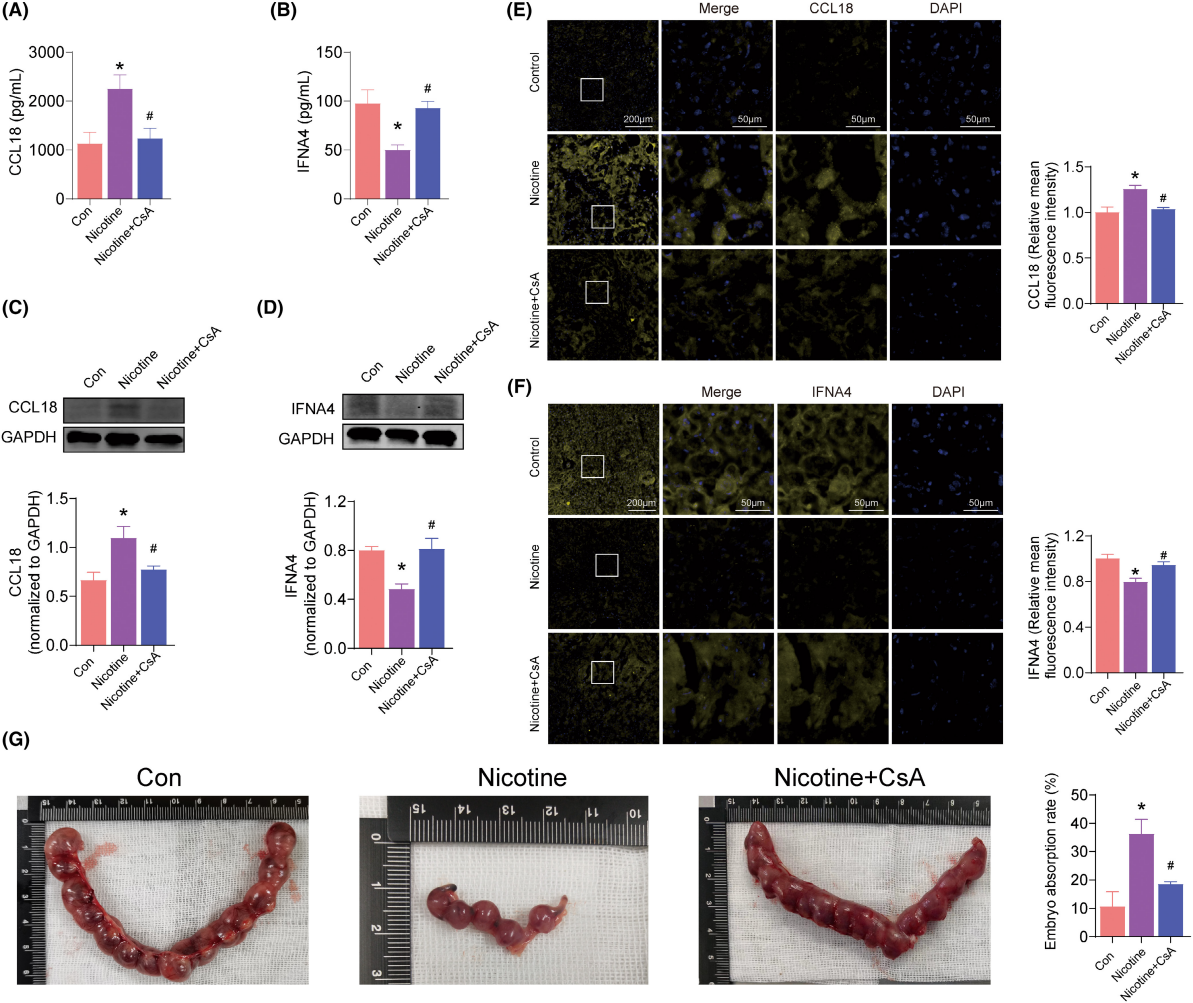
Validation of signature genes and potential therapeutic drugs in animals. (A, B) ELISA results of serum CCL18 and IFNA4 in mice. (C, D) Western blot results of CCL18 and IFNA4 in mice placenta. (E, F) Immunofluorescence results of CCL18 and IFNA4 in mice placenta. (G) The embryo absorption rate of different group. *p < 0.05 vs. Control group, #*p* < 0.05 vs. Nicotine group, by repeated‐measures one‐way anova followed by post hoc Dunnett's multiple comparisons test. All scale bars are 200 μm (E, F left) and 50 μm (E, F right).

The model of C57BL mice with or without PTE was established. In addition, the result showed that CCL18 was significantly increased (*p* < 0.05), and IFNA4 was remarkably decreased (*p* < 0.05), both in peripheral blood (Figure 11A,B) and in placenta of mice with PTE (Figure 11C–F). In addition, the embryo absorption rate in mice with PTE was significantly increased compared with the normal group (*p* < 0.05) (Figure 11G). This indicated that the concentration of these two molecules detected from peripheral blood can reflect the changes in placenta, which could noninvasively diagnosis of pathological changes in placenta due to PTE. Moreover, CsA significantly improved the levels of CCL18 and IFNA4 in PTE mice (*p* < 0.05) and reduced the level of embryo absorption rate (*p* < 0.05) (Figure 11A–G), which could be a potential drug to improve placental immune damage due to PTEd.

## 4 DISCUSSION

This current study initially disclosed the intimate association between PTE and immunological dysfunctions in the placenta, applying GSEA and immune enrichment examination. This led to the identification of 52 immune‐related DEGs, from which 12 pivotal genes, such as *JUN, FGF13* and *NR1I2*, were discerned via the MCC algorithm within the PPI network. These genes played a paramount role in protein interaction, mediating intricate signal intercommunication under PTE circumstances. To further comprehend the immune disturbance instigated by these hub genes in the presence of PTE, we meticulously explored the immune localization, physicochemical properties and molecular functions of the proteins encoded by these genes. It was found that the hub genes were primarily expressed in immunocytes and implicated in the regulation of immune function. As an exemplar, NR1I2 encodes the Pregnane X Receptor (PXR), a crucial modulator of xenobiotic and detoxification responses. Prior research confirmed that PXR could modulate Nuclear Factor κ‐Light‐Chain‐ Enhancer of B cells (NFκB) activation and the expression of numerous inflammatory mediators, thus precipitating aberrant tissue functionality.^44^, ^45^ However, its immunomodulatory role in the placenta remains uncharted. This investigation is the first to unveil the impact of NR1I2 on placental immune functionality under PTE. Additionally, we discovered a high affinity between nicotine, nitrosamine and NR1I2 through molecular docking analysis, possibly establishing one of the prerequisites for NR1I2 to instigate immune signal transduction under PTE conditions. In essence, probing the functions of these hub genes aids in comprehending the immunological implications of tobacco exposure on placental function.

Then, to delve further into the repercussions of PTE on the immune landscape within the placenta, an immune infiltration analysis was conducted on subtypes derived from consensus clustering of PTE samples. These subtypes were based on the 52 immune‐related genes previously identified. The ensuing findings disclosed notable shifts in both intrinsic immunocytes (M2 macrophages, neutrophils, resting NK cells) and adaptive immunocytes (follicular helper T cells, CD8+ T cells) when juxtaposed with control samples. Among these, NK cells and macrophages—comprising approximately 70% and 20%–30% of decidual leukocytes in the first trimester, respectively—predominate at the maternal‐foetal Interface.^46^, ^47^ NK cells exert significant influence over trophoblast invasion, spiral artery remodelling and placental development,^48^, ^49^ while macrophages play a pivotal role in implantation, trophoblast invasion, angiogenesis and pathogen clearance. These macrophages can be further divided into classically activated (M1) and alternatively activated (M2) subtypes.^50^ However, an excess of NK cells and an imbalanced M1/M2 macrophage ratio are linked to various pregnancy complications.^51^ Research by Prins JR et al.^11^ revealed a substantial surge in the proportion of decidual NK cells and a markedly higher M1/M2 macrophage ratio in smokers relative to non‐smokers, an observation that was intensified in heavy smokers. Similarly, Luppi P et al.^52^ confirmed a significant decline in CD56+ NK cells in the peripheral blood of pregnant smokers compared to non‐smoking expectant women of equivalent gestational age (14–20 weeks). Additionally, Belhareth et al.^53^ provided evidence that cigarette extracts influenced placental macrophage differentiation into multinucleated giant cells in a dose‐responsive manner, while simultaneously compromising critical placental macrophage functions such as granule uptake, surface marker expression, cytokine release and metalloproteinase gene expression. Synthesizing these findings with our own research, we propose that NK cells and macrophages play a significant role in immune disruptions within the placenta triggered by PTE. In the adaptive immunity, follicular T‐helper cells serve as an illustrative example. These recently identified CD4+ T‐helper cells are known to interact intensively with B lymphocytes, mediating their proliferation and activation.^54^ Zeng et al.^55^ have elucidated the role of uterine and placental fTh‐like cells within an allogenic‐normal‐pregnant mouse model. Concurrently, research has substantiated that patients with recurrent miscarriages present a significant surge in memory fTh cells, and demonstrate a robust correlation with B cells in decidual tissue.^56^ Furthermore, Heydarlou et al.^57^ uncovered that the frequency of circulating fTh cells was markedly elevated in women with preeclampsia compared to healthy controls. Yet, the impact of prenatal tobacco exposure on placental fTh cell infiltration remains inadequately researched. By integrating these insights with our findings, we conjecture that an increase in fTh cells within placental tissue due to prenatal tobacco exposure could be a pivotal driver of detrimental pregnancy outcomes such as recurrent miscarriages and preeclampsia. Nevertheless, the precise molecular mechanisms warrant further exploration.

To further explore the molecular mechanisms underlying immunocyte disruption in PTE, a Spearman correlation analysis was employed, leading to the identification of six genes, namely *FGR, ANGPT1, TEK, IFNAR2, CXCR4* and *IL1R1*. Among them, *IL1R1* demonstrated a significant correlation with natural killer (NK) cells in subtype C. Existing research substantiates IL1R as a critical mediator facilitating IL‐1‐dependent activation through its binding to IL‐1α and IL‐1β,^58^ thus inducing NK cells to secrete interferon, further intensifying the embryonic rejection effect of Th1 cells.^59^ Moreover, IL1R initiates IL‐1‐dependent signalling pathways in the uterus, including NF‐kappa‐B and MAPK, which have been demonstrated to regulate the proliferation, activation and apoptosis of NK cells.^60^ This accords with the enrichment analysis of genes in the green module, which was tightly associated with NK cells derived from WGCNA. Beyond the NF‐kappa‐B and MAPK pathways, these genes also showed enrichment in the JAK–STAT signalling pathway and the Wnt signalling pathway. Published studies corroborate that STAT3 knockdown in NK cells correlates with a diminished proliferation rate, while STAT3 overexpression augments human NK cell expansion.^61^, ^62^, ^63^ Furthermore, the Wnt signalling pathway has been demonstrated to have implications for the development and activation of NK cells.^64^ Collectively, the integration of Spearman correlation analysis, WGCNA, and the enrichment analysis of genes in key modules offers comprehensive insights into the pathophysiological mechanisms underlying abnormal immunocyte phenotypes and functions resultant from PTE.

Molecular contact on the membrane surface represents the primary mode of communication between immunocytes. Given the enrichment of DEGs related to immune function, chiefly in the cytokine‐cytokine receptor interaction, we scrutinized the ligand–receptor attributes of these 52 DEGs. This exploration facilitated the construction of a network that incorporated variably infiltrated immune cells, thereby elucidating potential intercellular communication mechanisms attributable to PTE. Our investigation suggested that a considerable portion (20 out of 52) of the immune‐related DEGs served as ligands or receptors, facilitating cross‐talk with variably infiltrated immune cells such as CD8+ T cells, neutrophils, macrophages and NK cells. For instance, platelet factor 4 (PF4) and protein C receptor (PROCR) constituted significant nodal points of interaction among these cells. Previous research has verified the role of PF4, secreted by the alpha granules of activated platelets, in exerting chemotactic influences on several cell types and acting as an inhibitor of haematopoiesis, angiogenesis and T‐cell function.^65^ Concurrently, PROCR has been deemed critical in vascular establishment during embryonic development.^66^, ^67^ Furthermore, both macrophages and NK cells have been documented as significant contributors to placental angiogenesis.^68^, ^69^ From these observations, we postulated that PTE affected the synergistic effect of immune cells in angiogenesis, as orchestrated by PF4 and PROCR. Additional ligand–receptors involved in this intercellular communication network afford valuable insights into alterations in the immune microenvironment. Collectively, these analyses implied that PTE‐induced differential expression of hub genes could cause cellular communication to be disrupted and cause pathological immuno‐infiltration characteristics.

Concerning diagnosis, the significant alterations in gene expression profiles due to PTE present considerable obstacles for conventional research methodologies to discern sensitive biomarkers capable of promptly identifying placental immune abnormalities. This difficulty precipitates delays in preemptive measures. The integration of transcriptome microarray‐based bioinformatics and machine learning offers an optimal solution to navigate this impasse, markedly enhancing diagnostic model precision. In the present investigation, univariate logistic regression, LASSO, RF and ANN algorithms identified 11 signature genes. Subsequent testing sets, incorporating peripheral blood samples with and without PTE as well as animal experiments, provided further validation for CCL18 and IFNA4. Of these, CCL18 has been identified as a macrophage differentiation factor promoting an M2 spectrum macrophage phenotype.^70^, ^71^, ^72^ Contemporary research recognizes CCL18 as an immune‐related sensitive predictor for preeclampsia.^73^ Regarding IFNA4, primarily produced by macrophages, it participates extensively in several immune‐related processes, encompassing lymphocyte activation, immune responses and antiviral activities.^74^ It has been confirmed that IFNA4 is significantly overexpressed in male intrauterine growth restricted (IUGR) rats and has a close association with multiple inflammatory pathways.^75^ Research exploring the correlation between CCL18, IFNA4 and PTE remains limited. Considering the established functions of CCL18 and IFNA4, we postulate a potential correlation with the placenta's diminished capacity to resist infectious agents attributable to PTE.^76^, ^77^ For the first time, our study underscores the predictive utility of CCL18 and IFNA4 concerning placental immune disruption due to PTE. Additionally, we substantiated the differential expression of CCL18 and IFNA4 in the peripheral blood of PTE‐affected animals, mirroring changes evident in placental tissue. Of course, a comprehensive validation of our findings in a large‐scale controlled study is yet to be undertaken. Furthermore, in light of peripheral blood RNA and protein's inherent instability, the exploration for more innovative, sensitive and stable biomarkers (such as miRNAs or exosome) continues. Importantly, we also identified 34 compounds potentially targeting key representative genes to mitigate PTE‐induced placental immune disorders, including oestradiol, progesterone and Cyclosporin A (CsA). As an effective immunosuppressant, CsA is broadly employed in organ transplantation.^78^ Now, CsA is also extensively utilized in various pregnancy complications, such as idiopathic or recurrent spontaneous abortion,^79^, ^80^ repeated implantation failure^81^, ^82^ and preeclampsia.^83^ This therapeutic effect is supposed to be attributed to its widespread modulatory effects on various immunocytes. Existing research indicates that CsA can influence T cells, NK cells and macrophages^84^, ^85^, ^86^ precisely the cells disrupted by PTE. Hence, we propose a potential therapeutic role for CsA in PTE cases. Nonetheless, no research to date has elucidated CsA's protective role in placental immune disorders secondary to PTE. Our discovery may extend the clinical applications of CsA, offering a potentially viable treatment alternative for patients experiencing PTE.

## 5 CONCLUSION

Despite a substantial number of women experiencing PTE, our grasp of how such exposure precipitates pathologic pregnancy outcomes remains relatively nascent. Given the intimate relationship between the placental immune microenvironment and foetal development, an exhaustive exploration of how PTE modulates placental immune regulation holds transformative potential for timely intervention and treatment. Our study, the first to delve into PTE's impact on the placental immune landscape at molecular and systemic levels, employs an integrated approach of bioinformatics, machine learning and animal experimentation. We have established that one salient pathological characteristic under PTE is immune disruption, evidenced by significant alterations in numerous immune‐related genes, molecular signalling pathways, and the prevalence and function of placental immunocytes. Moreover, our research not only elucidates these pathological modifications but also examines the interconnections between these variations, offering novel perspectives to interpret the pathogenesis linked with adverse pregnancy outcomes due to PTE, and charting a pioneering trajectory for studying PTE‐induced placental immune disorders. Furthermore, we are the first to pinpoint sensitive peripheral blood biomarkers capable of reflecting placental immune disorders, achieved through machine learning and animal testing, while also investigating potential compounds capable of intervening in these immune disorders. In essence, our work proposes an effective strategy to preempt and counteract pathological pregnancy outcomes due to tobacco exposure, significantly bridging the current research chasm in this field. We anticipate our research will inspire additional studies in this domain. Nonetheless, our study is not without limitations. First, our results primarily hinge on bioinformatics analysis, and although validated with an external test dataset and animals, these results still warrant further in vitro and clinical experiments for accuracy confirmation. Second, the immune infiltration analysis employed the widely used CIBERSORT algorithm for immunocyte quantification, but single‐cell sequencing remains necessary for the most accurate data. Finally, while we identify a correlation between DEGs and abnormal immune microenvironment, the cause–effect relationship between the two necessitates further exploration. In conclusion, our results affirm the intimate association of PTE with placental immune microenvironment disorders, contributing new insights into understanding the pathological mechanisms, diagnosis and treatment of adverse pregnancy outcomes consequent to PTE.

## AUTHOR CONTRIBUTIONS

**Xiaoxuan Zhao:** Conceptualization (equal); data curation (equal); formal analysis (equal); software (equal); writing – original draft (equal). **Yuepeng Jiang:** Data curation (equal); formal analysis (equal); methodology (equal); project administration (equal). **Xiao Ma:** Investigation (equal); methodology (equal); validation (equal). **Qujia Yang:** Funding acquisition (equal); methodology (equal); software (equal). **Xinyi Ding:** Data curation (equal); formal analysis (equal); resources (equal). **Hanzhi Wang:** Formal analysis (equal); investigation (equal). **Xintong Yao:** Data curation (equal); formal analysis (equal); validation (equal). **Linxi Jin:** Data curation (equal); methodology (equal); software (equal). **Qin Zhang:** Data curation (equal); formal analysis (equal); supervision (equal); writing – review and editing (equal).

## FUNDING INFORMATION

This research was funded by National Natural Science Foundation of China (82305294); TCM Science and Technology Project of Zhejiang Province (2023ZR038); 2022 Research Project of the Affiliated Hospital of Zhejiang Chinese Medical University (2022FSYYZQ16); Hangzhou Health Science and Technology Project (A20230675) to X.X.Z.; China Postdoctoral Science Foundation (2023M733193) to Y.P.J.; Hangzhou Health Science and Technology major project (Z20230103) to Q.Z.

## CONFLICT OF INTEREST STATEMENT

The authors declare no conflict of interest.

## INFORMED CONSENT STATEMENT

Not applicable.

## Supporting information


Figure S1.

Figure S2.

Figure S3.

Figure S4.

Figure S5.

Table S1.

## Data Availability

The original contributions presented in the study are included in the article/supplementary material, further inquiries can be directed to the corresponding authors.
