# Supplementary figures and images for: Demystifying the impact of prenatal tobacco exposure on the placental immune microenvironment: Avoiding the tragedy of mending the fold after death
Source: J Cell Mol Med. 2023 Sep 12;27(20):3026–52. doi: 10.1111/jcmm.17846 (PMC10568673; doi:10.1111/jcmm.17846)

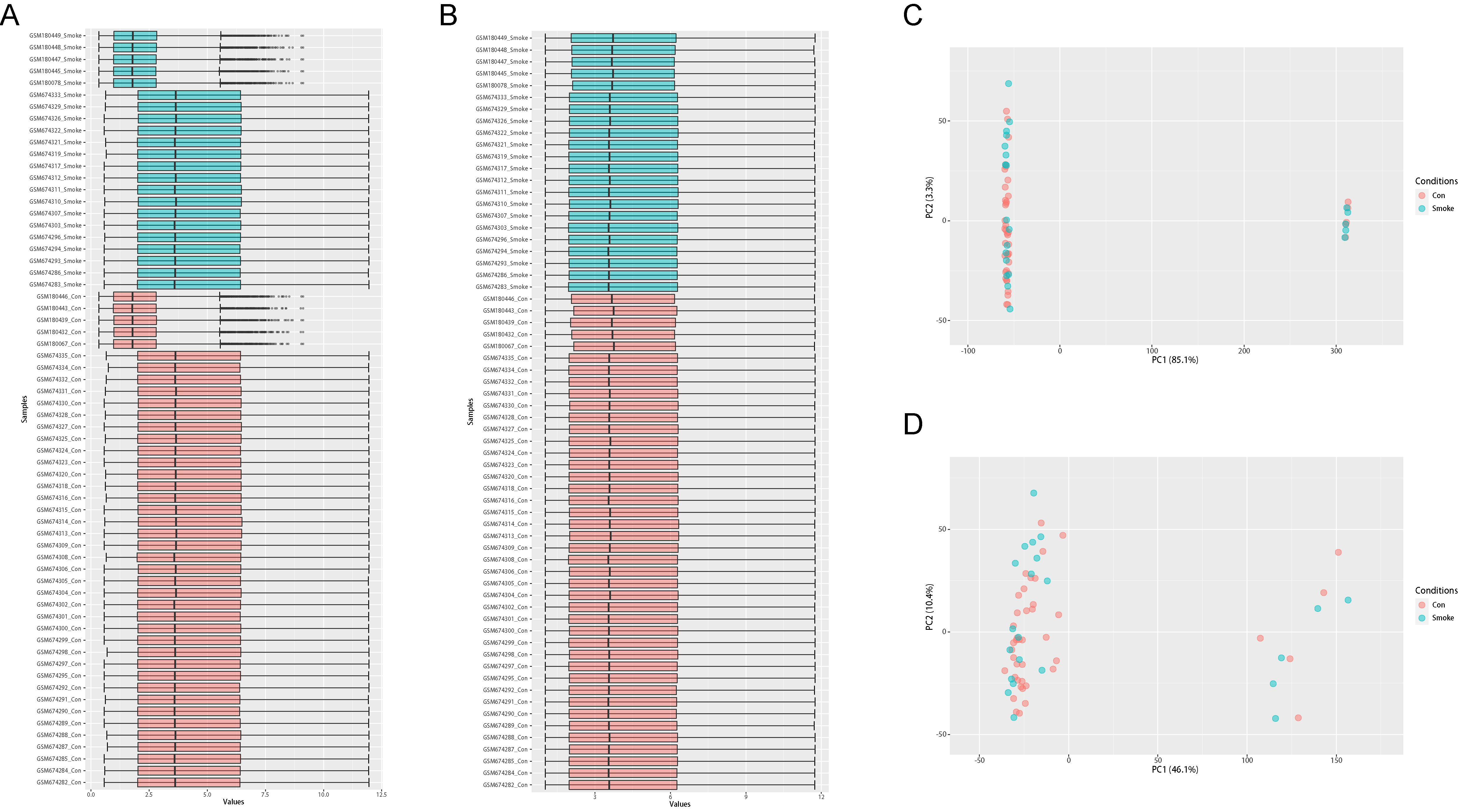

Supplement: Supplementary file 1 — Figure S1. [file JCMM-27-3026-s006.tif]

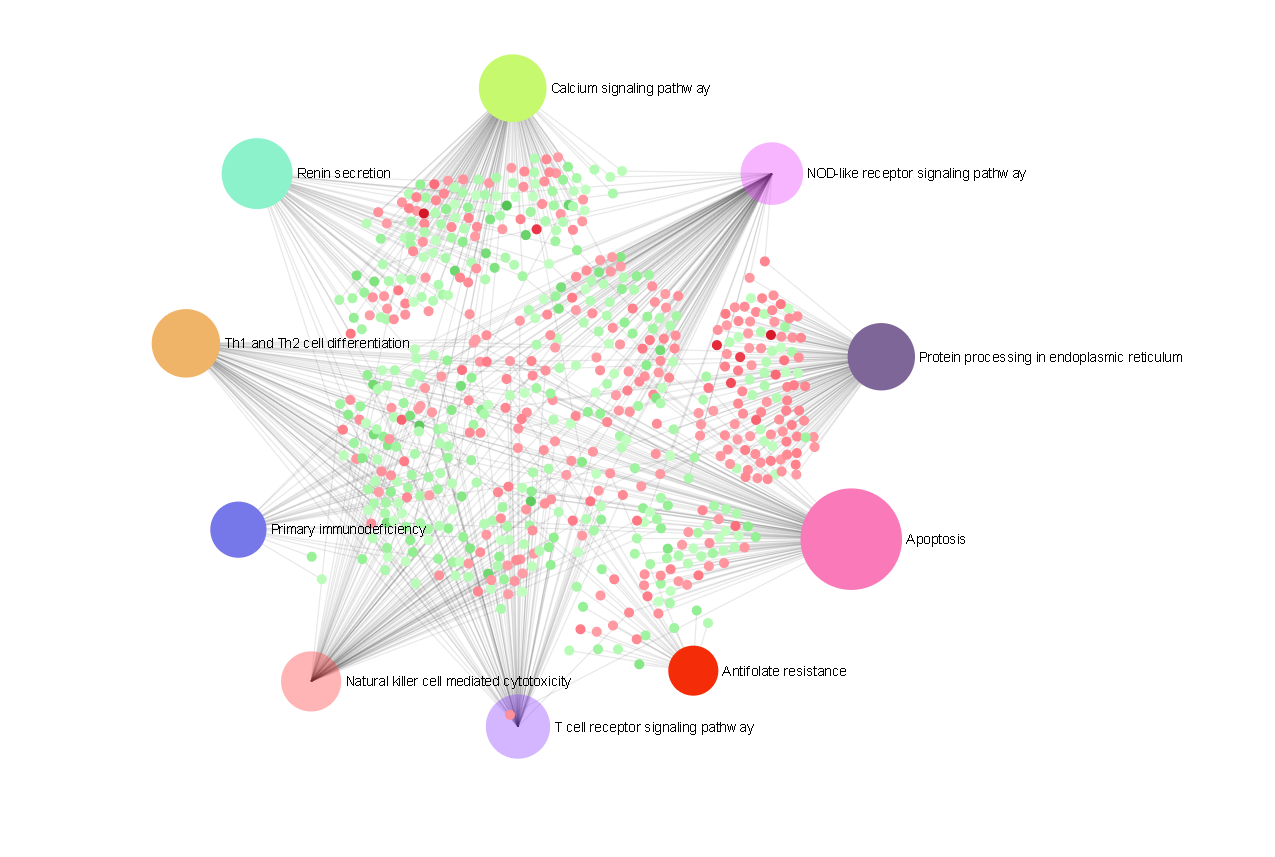

Supplement: Supplementary file 2 — Figure S2. [file JCMM-27-3026-s001.tif]

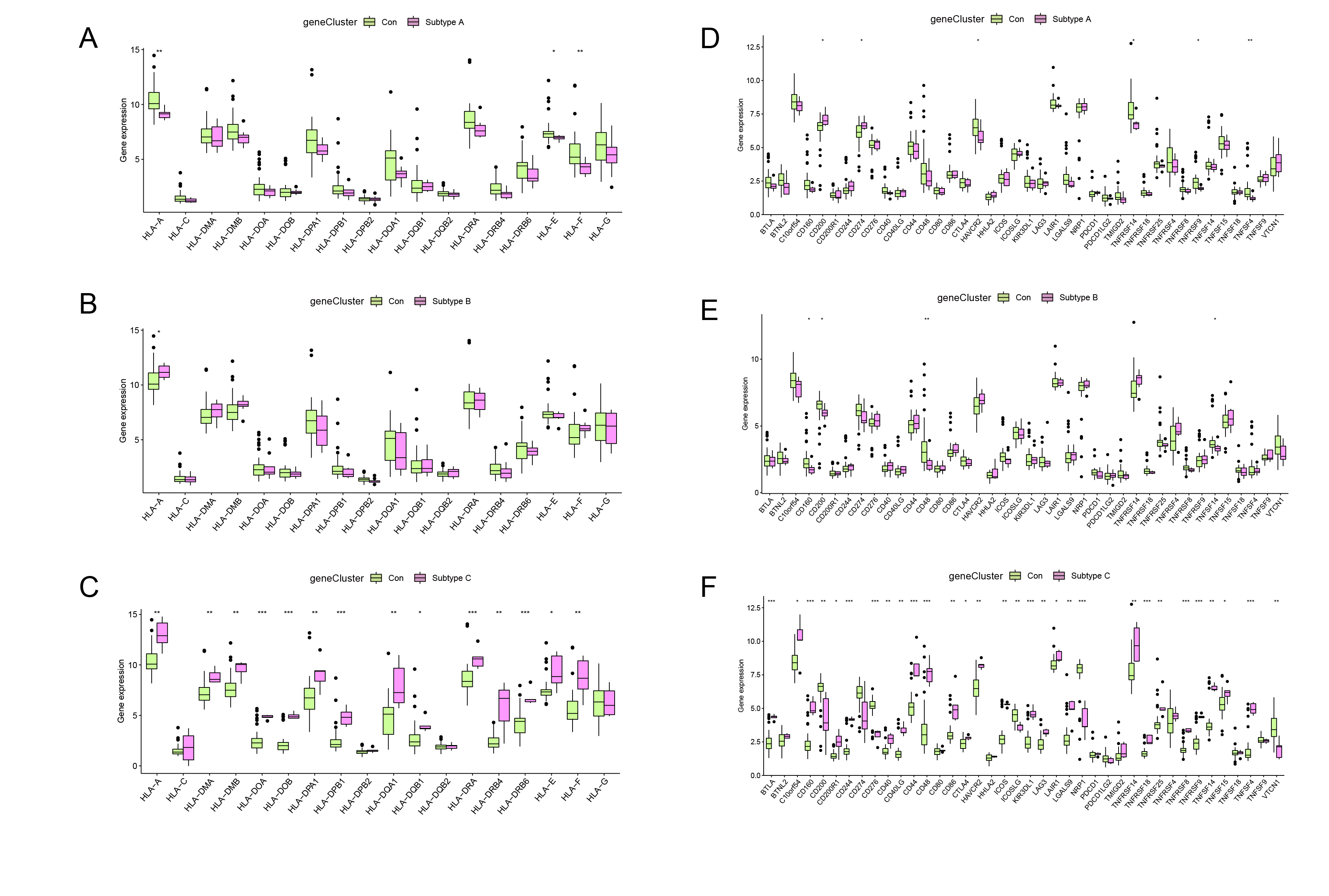

Supplement: Supplementary file 3 — Figure S3. [file JCMM-27-3026-s003.tif]

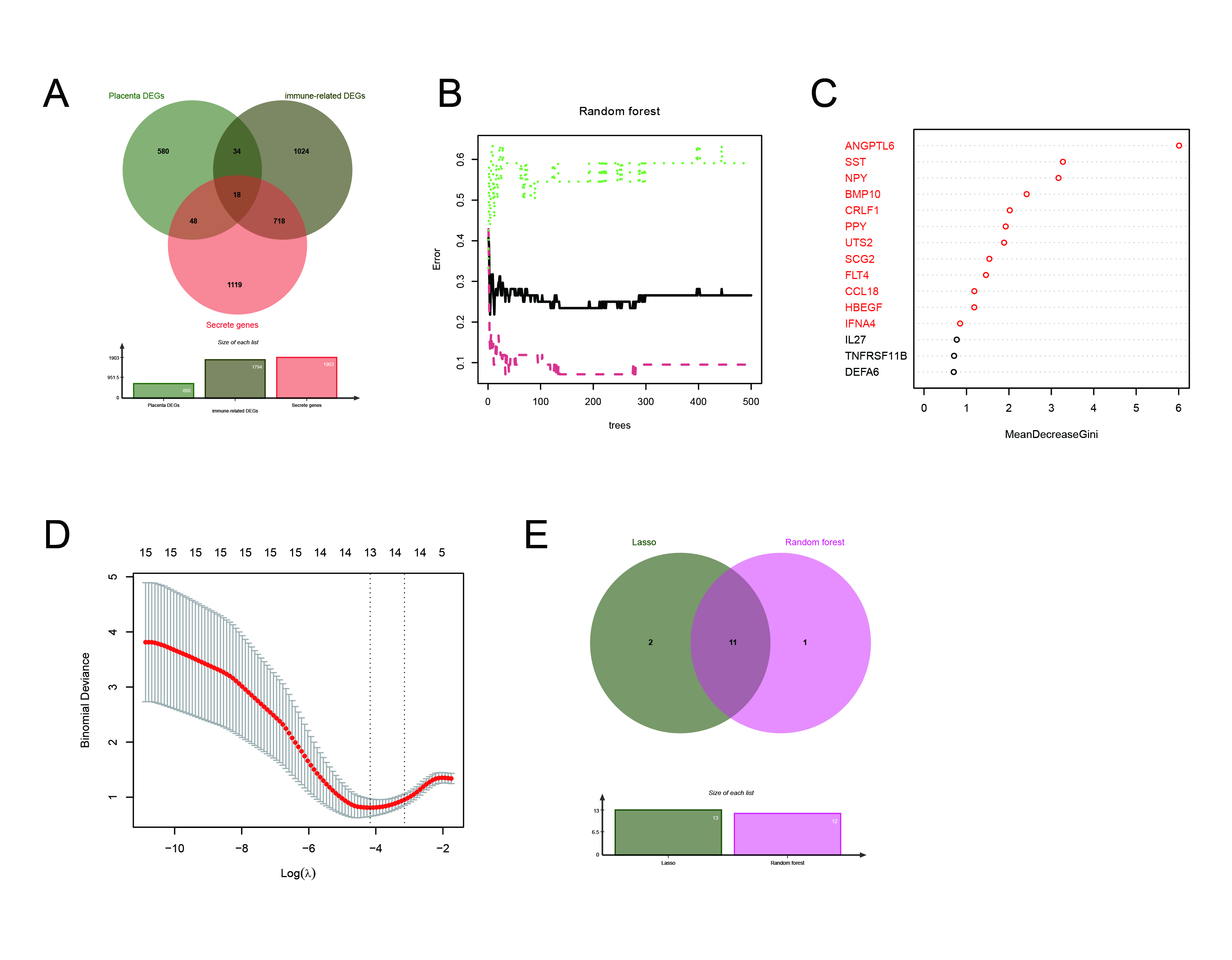

Supplement: Supplementary file 4 — Figure S4. [file JCMM-27-3026-s002.tif]

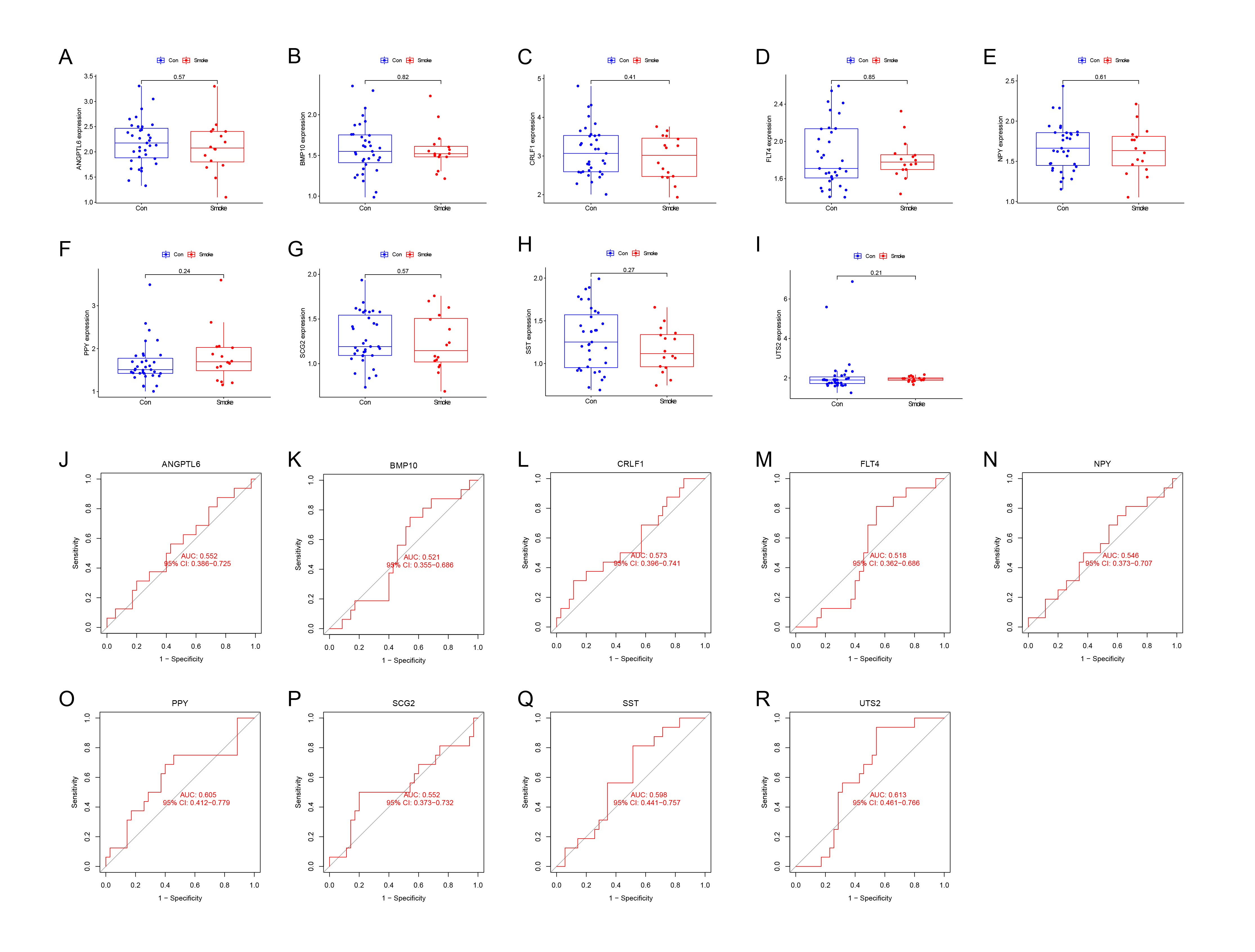

Supplement: Supplementary file 5 — Figure S5. [file JCMM-27-3026-s005.tif]
