# Supplementary material for: Demystifying the impact of prenatal tobacco exposure on the placental immune microenvironment: Avoiding the tragedy of mending the fold after death
Source: J Cell Mol Med. 2023 Sep 12;27(20):3026–52. doi: 10.1111/jcmm.17846 (PMC10568673; doi:10.1111/jcmm.17846)
Supplement: Supplementary file 6 — Table S1. [file JCMM-27-3026-s004.doc]

Supplementary Table 1. One-way logistic regression analysis on the 18 genes

| Genes | *P* | Odds Ratio |
| --- | --- | --- |
| SST | 0.009 | 26.311(2.279-303.818) |
| IFNA4 | 0.013 | 0.061(0.007-0.549) |
| UTS2 | 0.014 | 37.615(2.053-689.208) |
| HBEGF | 0.016 | 2.239(1.165-4.306) |
| FLT4 | 0.019 | 0.571(0.357-0.912) |
| IL27 | 0.024 | 0.359(0.147-0.875) |
| PPY | 0.025 | 8.476(1.301-55.231) |
| ANGPTL6 | 0.031 | 2.583(1.093-6.103) |
| CCL18 | 0.034 | 4.809(1.122-20.619) |
| NPY | 0.034 | 12.133(1.203-122.398) |
| DEFA6 | 0.042 | 9.438(1.09-81.708) |
| BMP10 | 0.043 | 0.133(0.019-0.942) |
| SCG2 | 0.044 | 2.402(1.026-5.625) |
| TNFRSF11B | 0.048 | 2.903(1.009-8.355) |
| CRLF1 | 0.049 | 2.279(1.004-5.176) |
| UCN | 0.051 | 0.458(0.208-1.005) |
| PF4 | 0.054 | 6.306(0.967-41.102) |
| SFTPD | 0.055 | 2.565(0.979-6.72) |
